# Iduronate Ring Puckering Effects on Preferred Glycosidic Linkage Conformations in Heparin/Heparan Sulfate and Dermatan Sulfate Disaccharides

**DOI:** 10.3390/molecules31030504

**Published:** 2026-02-02

**Authors:** Olgun Guvench

**Affiliations:** Department of Pharmaceutical Sciences and Administration, School of Pharmacy, Westbrook College of Health Professions, University of New England, 716 Stevens Avenue, Portland, ME 04103, USA; oguvench@une.edu

**Keywords:** iduronate, IdoA2S, glycosaminoglycan, glycosidic linkage, ring pucker, molecular dynamics, free energy, biased sampling, adaptive biasing force, CHARMM

## Abstract

The conformation of a glycosaminoglycan (GAG) carbohydrate biopolymer is dependent upon the ring puckering states of its constituent monosaccharide residues and the dihedral angles (*φ*, *ψ*) of the glycosidic linkages connecting these residues. In the context of GAGs, the monosaccharide residue iduronate (IdoA; the conjugate base of iduronic acid) is able to take on both chair and boat-like ring pucker states. All-atom explicit-solvent molecular dynamics simulations were applied to determine the extent to which IdoA ring pucker state affects the conformational preferences of (*φ*, *ψ*) in 16 different IdoA-containing disaccharides derived from the GAGs heparin/heparan sulfate and dermatan sulfate. Using the extended-system adaptive biasing force (eABF) method, the complete free-energy surface Δ*G*(*φ*, *ψ*) was computed for each disaccharide with its IdoA ring restrained separately to the ^1^C_4_, ^2^S_O_, B_3,O_, or ^4^C_1_ ring pucker state. Global-minimum Δ*G*(*φ*, *ψ*) values resided within broad Δ*G*(*φ*, *ψ*) basins, and both ring pucker state and sulfation status influenced basin shape and size. Various sulfoforms of the disaccharide IdoA*α*1–4GlcNS had prominent secondary-minimum basins distinct from the global-minimum basins, and these secondary-minimum basins may manifest as metastable states in standard (nonbiased) molecular dynamics simulations on the 1-microsecond timescale. As such, the present results provide a reference for assessing (*φ*, *ψ*) sampling in nonbiased molecular dynamics simulations of GAGs and demonstrate the interplay between IdoA ring puckering, glycosidic linkage dihedral rotation, and sulfation status in contributing to GAG conformational preferences.

## 1. Introduction

**Figure 1:**
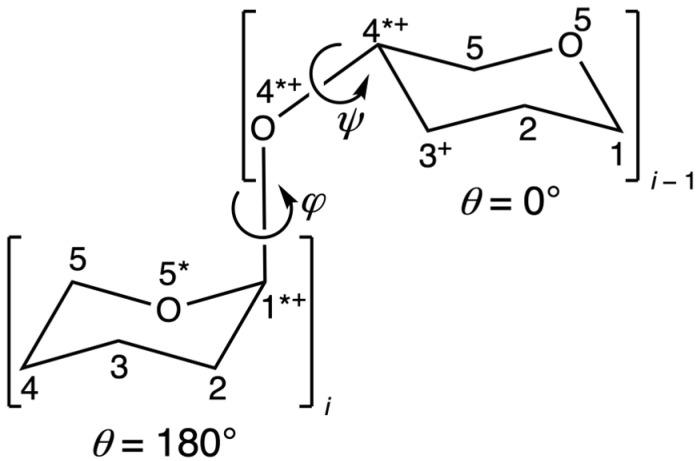
CP *θ* puckering parameters and glycosidic linkage (*φ*, *ψ*) dihedrals for IdoA*α*1–4Glc disaccharide. Brackets indicate monosaccharide residues, residue *i* corresponds to IdoA, and residue *i* − 1 corresponds to Glc; exocyclic functional groups have been omitted for clarity. Residue *i* is drawn in the *θ* = 180° ^1^C_4_ chair pucker state and residue *i* − 1 is drawn in the *θ* = 0° ^4^C_1_ chair pucker state; the CP parameter *ϕ* is not relevant for chair pucker states. Carbon and oxygen atoms have been numbered per IUPAC conventions [10], “*” indicates an atom belonging to the *φ* dihedral, and “+” indicates an atom belonging to the *ψ* dihedral.

Glycosaminoglycans (GAGs) are linear (non-branched) carbohydrate biopolymers composed of disaccharide repeats [1]. GAGs function as both structural and signaling molecules, and these functions depend upon their chemical compositions as well as their three-dimensional (3D) conformations [2,3,4,5]. Two chief determinants of GAG 3D conformation are the ring pucker states of the constituent monosaccharide residues and rotation about the dihedral angles in the glycosidic linkages that connect the residues [6,7]. Ring puckering is conveniently represented by the Cremer–Pople (CP) parameters (*θ*, *ϕ*) [8], which are functions of the ring dihedrals, while the dihedral angles (*φ*, *ψ*) of the glycosidic linkage connecting residues *i* and *i* − 1 are defined by IUPAC as *φ* ≡ O5*_i_*–C1*_i_*–OX*_i_*_−1_–CX*_i_*_−1_ and *ψ* = C1*_i_*–OX*_i_*_−1_–CX*_i_*_−1_–C(X − 1)*_i_*_−1_, where X = 3 for a 1–3 linkage and X = 4 for a 1–4 linkage [9] (*n.b.*: in NMR studies of carbohydrates, hydrogen atoms typically replace O5*_i_* and C(X − 1)*_i_*_−1_ in dihedral angle definitions). Figure 1 illustrates CP parameters and (*φ*, *ψ*) in the context of the disaccharide IdoA*α*1–4Glc, where IdoA is L-iduronate and Glc is D-glucose.

**Figure 2:**
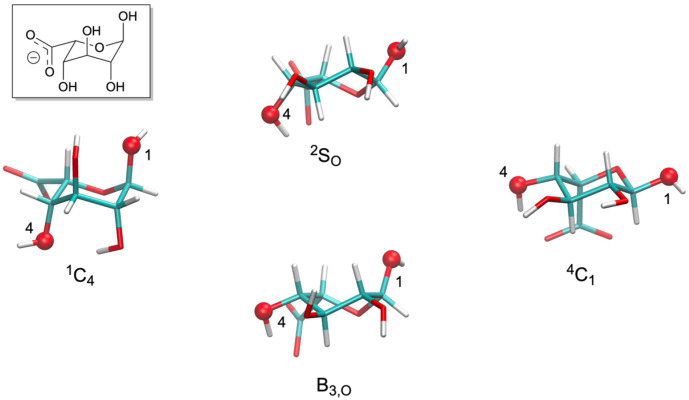
IdoA ring puckering. ^1^C_4_, ^2^S_O_, B_3,O_, and ^4^C_1_ pucker states are shown, with O1 and O4 atoms rendered as spheres and labeled. The “^2^S_O_/B_3,O_” pucker state is an intermediate conformation between the ^2^S_O_ and B_3,O_ pucker states. Inset: chemical structure of IdoA. All structures here have the *α*-anomeric stereochemistry at C1.

Unlike other monosaccharide components of GAGs that tend to be thermodynamically confined to the ^4^C_1_ pucker state, iduronate (IdoA) and its 2-*O*-sulfated form (IdoA2S) are able to access multiple puckering states under typical biological conditions [7,11,12,13,14,15,16,17] (*n.b.*: in what follows, “IdoA[2S]” indicates that the residue may be either the non-sulfated or the 2-*O*-sulfated form). Specifically, O1-substituted IdoA is engaged in a three-way equilibrium between ^1^C_4_, ^2^S_O_/B_3,O_, and ^4^C_1_ pucker states, where “^2^S_O_/B_3,O_” indicates a pucker state that is intermediate between ^2^S_O_ and B_3,O_, and O1-substituted IdoA2S is engaged in a three-way equilibrium between ^1^C_4_, B_3,O_, and ^4^C_1_ pucker states (Figure 2) [16]. O1,O4-disubstituted IdoA[2S], as occurs in GAGs, is engaged in a two-way equilibrium between ^1^C_4_ and ^2^S_O_ pucker states without significant contribution from ^4^C_1_ or B_3,O_ pucker sampling [16]. As such, the 3D conformations of the IdoA[2S]-containing GAGs heparin/heparan sulfate (HS) and dermatan sulfate (DS) may depend on both (*φ*, *ψ*) and ring puckering. Heparin has important clinical value as an anticoagulant and antithrombotic [18], and proper treatment of pucker states is required in order to avoid misleading conclusions from computational studies of heparin-containing systems [19]. Interest in HS and DS stems from their roles as major components of proteoglycans (PGs), wherein they are covalently conjugated to a protein core and contribute to both structural and signaling functions of PGs [20,21,22].

The present work explores the interplay between IdoA[2S] pucker state and glycosidic linkage conformational preferences. IdoA[2S] pucker state transitions occur on the microsecond timescale [23], which necessitates standard (nonbiased) molecular dynamics trajectories on the order of 10 microseconds for converged statistics, which is computationally demanding. The computational demand is compounded by the large variety of sulfoforms of heparin/HS and DS, as these may have different balances of puckering preferences [16], and therefore would need to be independently simulated. Given this challenge, the approach taken here is to used biased sampling to quantitate the complete free-energy surface Δ*G*(*φ*, *ψ*) in IdoA[2S]-containing disaccharides as a function of IdoA[2S] pucker state. To this end, comprehensive all-atom explicit-solvent molecular dynamics with the CHARMM36 additive force field [24,25,26,27,28] and extended-system adaptive biasing force (eABF) [29,30] sampling were performed on 64 distinct heparin/HS and DS disaccharide systems, as distinguished by chemical composition (including sulfoform) and IdoA[2S] pucker state (restrained to ^1^C_4_, ^2^S_O_, B_3,O_, or ^4^C_1_).

Lutsyk and Plazinski’s recent results on the conformational properties of 106 GAG-related disaccharides [31] are the most directly related to the present effort, including the identities of the disaccharides, the choice of force field, the use of explicit solvent, and the application of biased molecular dynamics to achieve mapping of the complete free-energy surface Δ*G*(*φ*, *ψ*). While the IdoA ring remains ^1^C_4_ throughout their disaccharide simulations, the authors provide supplementary examples of Δ*G*(*φ*, *ψ*) as a function of two different IdoA ring pucker states for four disaccharides that demonstrate modest changes in the shapes and sizes of global-minimum Δ*G*(*φ*, *ψ*) basins as a function of IdoA pucker. This effect of ring pucker state on Δ*G*(*φ*, *ψ*) is in line with observations from the same group for uniform pentasaccharides composed of Glc or one of its seven stereoisomers formed by epimerization at C2, C3, and/or C4 [32]. It also correlates with experimental work supporting the view that “[Heparin polymer IdoA] ^1^C_4_ and ^2^S_O_ conformers may interconvert with little change to the geometry of the glycosidic linkages to adjacent residues in the polysaccharide chain [33].”

The results of the present work show that IdoA pucker state as well as the disaccharide sulfoform can influence Δ*G*(*φ*, *ψ*). While the changes in Δ*G*(*φ*, *ψ*) are not “dramatic” in the sense that the global minimum does not shift from one quadrant of (*φ*, *ψ*) space to another as a function of IdoA[2S] pucker state, the changes are sufficient to yield qualitative changes in the spatial disposition of the non-reducing end of the disaccharide relative to the reducing end. Alignment of minimum-Δ*G*(*φ*, *ψ*) disaccharide conformations with identical chemical compositions but different pucker states demonstrates the resulting qualitative differences. When the alignment is performed using C1 and directly attached atoms at the reducing end of the compared disaccharides, the vector of the C–O bond that would form a glycosidic linkage at the non-reducing end can vary substantially both in direction and absolute position. Additionally, some heparin/HS-derived disaccharides in the set are seen to have a local Δ*G*(*φ*, *ψ*) minimum of less than +3 kcal/mol in a different quadrant of (*φ*, *ψ*) space than the quadrant that contains their global minimum. These minima are observed to be separated by free-energy barriers sufficiently high to create metastable states in 1-microsecond-timescale molecular dynamics simulations, and the locations of these local minima in (*φ*, *ψ*) space and the shapes and sizes of their associated Δ*G*(*φ*, *ψ*) basins shift depending upon IdoA[2S] pucker state.

## 2. Results and Discussion

**Figure 3:**
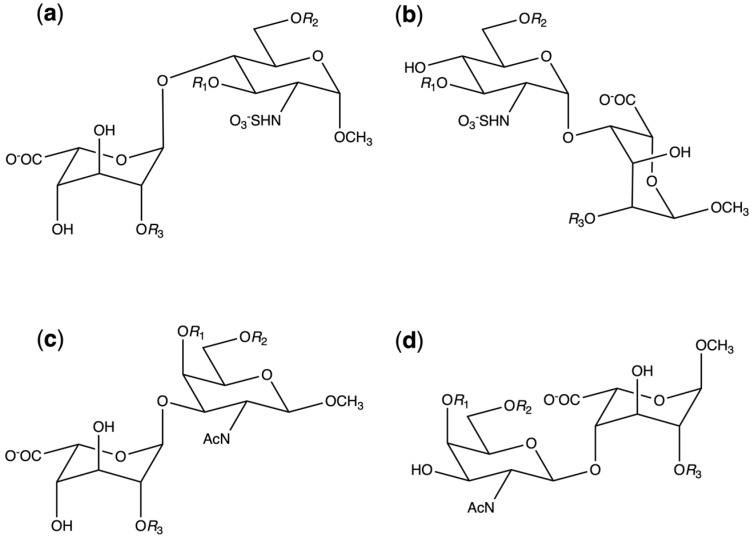
Heparin/HS and DS disaccharide templates. (**a**) **HS1*n***, (**b**) **HS2*n***, (**c**) **DS1*x***, and (**d**) **DS2*x***. –O*R*_1_, –O*R*_2_, and –O*R*_3_ may be –OH or –OSO_3_^−^ (Table 1).

Eight distinct disaccharides were considered for heparin/HS and for DS. For the purposes of the present work, they are abbreviated as follows: [**GAG**][**disaccharide type**][**sulfation pattern**], where [**GAG**] ∈ {**HS**, **DS**} and [**disaccharide type**] ∈ {**1, 2**}. For **HS**, [**sulfation pattern**] = ***n*** ∈ {**1, 2, 3, 4**}, and for **DS**, [**sulfation pattern**] = ***x*** ∈ {**a, b, d, e**}. [**GAG**][**disaccharide type**] combinations create disaccharide templates subject to variable sulfation as illustrated in Figure 3, with the [**sulfation pattern**] key listed in Table 1. For both **HS** and **DS** disaccharides, three hydroxyl positions are considered here for sulfation. If these are taken combinatorially, 2^3^ = 8 sulfoforms are possible for each disaccharide template; however, only four sulfoforms for each disaccharide template are examined in the present work since they are the biologically relevant species. For **HS** disaccharides, the degree of sulfation increases with ***n***: **1** < **2** < **3** < **4**, consistent with known biosynthetic pathways [1,34]. For **DS** disaccharides, ***x*** is assigned a letter value based on standardized nomenclature for the biological iA, iB, iD, and iE DS sulfoforms [22,35,36]. The iC DS sulfoform is not included because its existence has not been experimentally verified [37,38]. Table S1 in the Supplementary Materials contains a complete list of the abbreviations and chemical compositions of the disaccharides.

For each of these 16 disaccharides, the complete free-energy surface Δ*G*(*φ*, *ψ*) was computed separately for IdoA[2S] restrained to each of the ^1^C_4_, ^2^S_O_, B_3,O_, and ^4^C_1_ pucker states, for a total of 64 systems distinct in chemical composition and/or IdoA[2S] ring pucker, and with each system simulated in triplicate (see “Section 4”). The B_3,O_ and ^4^C_1_ pucker state simulations have been included for completeness, as these ring puckers may occur in the context of O1-substituted IdoA[2S] [16]; however, for O1,O4-disubstituted IdoA[2S], as occurs in GAG biopolymers, ring puckering is limited to ^1^C_4_ and ^2^S_O_, possibly with a minor fraction of ^4^C_1_ [16,39]. Therefore, the following discussion emphasizes the ^1^C_4_ and ^2^S_O_ results.

Only a single member of the triplicate Δ*G*(*φ*, *ψ*) dataset for each disaccharide is presented below, and these data are presented for only half the span of *φ* as the other half is typically associated with high values of Δ*G*(*φ*, *ψ*) consistent with the *exo*-anomeric effect [33]. The complete triplicate data demonstrate generally good convergence of Δ*G*(*φ*, *ψ*) across the full span of (*φ*, *ψ*) space, including the locations of Δ*G*(*φ*, *ψ*) = 0 kcal/mol (Table S2) and the shapes and sizes of global-minimum Δ*G*(*φ*, *ψ*) basins (Figure S1). In the 7 cases out of 64 disaccharide/pucker combinations where the location of Δ*G*(*φ*, *ψ*) = 0 kcal/mol is not in quantitative agreement amongst the triplicate data, Δ*G*(*φ*, *ψ*) either has two distinct Δ*G*(*φ*, *ψ*) ≈ 0 kcal/mol minima in the same quadrant of (*φ*, *ψ*) space or a broad global-minimum basin wherein a large region of (*φ*, *ψ*) space has Δ*G*(*φ*, *ψ*) ≈ 0 kcal/mol (Table S2). Shifts in the location of Δ*G*(*φ*, *ψ*) = 0 kcal/mol due to changes in pucker state are tabulated in Table S3.

**Figure 4:**
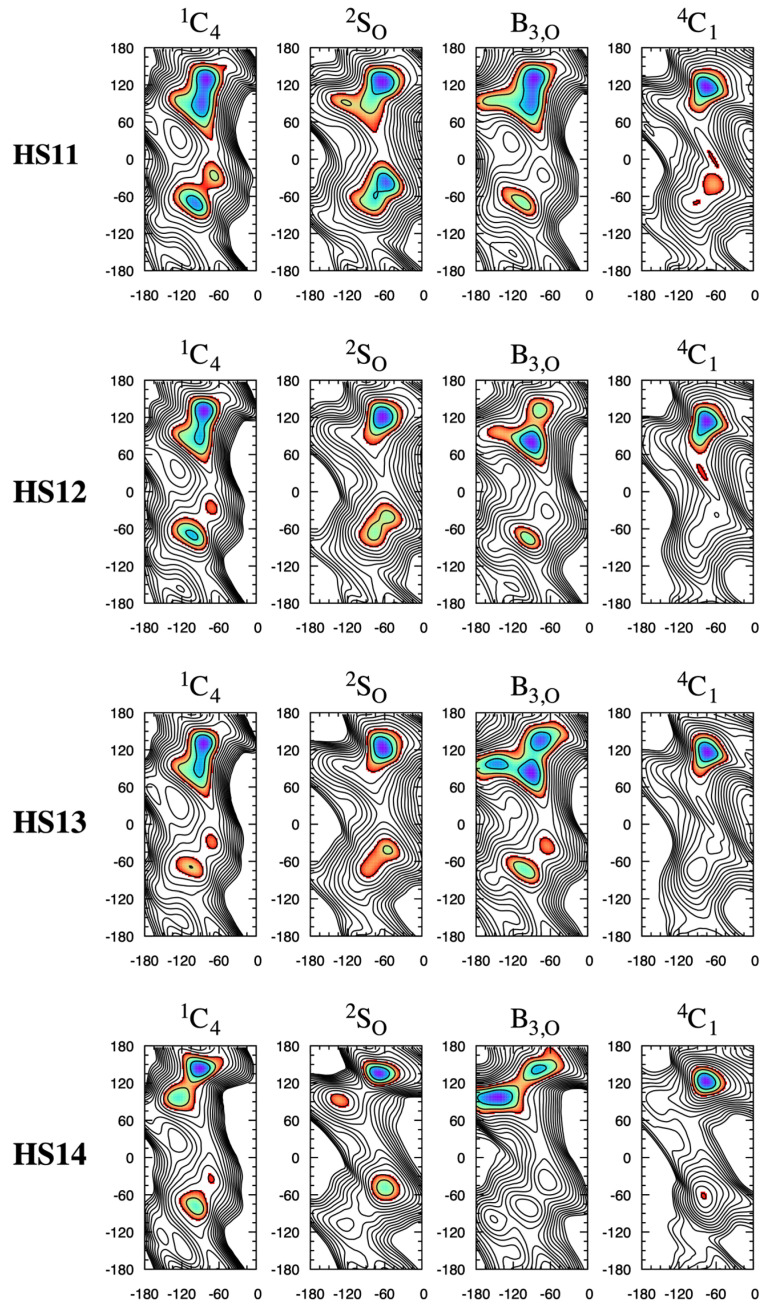
**HS1*n*** Δ*G*(*φ*, *ψ*) as a function of IdoA pucker. Each row is for a single disaccharide and each column is for a single IdoA pucker state. *φ* is on the *x*-axis and *ψ* is on the *y*-axis, with their values in degrees. Δ*G*(*φ*, *ψ*) is in kcal/mol with contours every 1 kcal/mol from 0 to 15 kcal/mol and is colored from blue to red in the range 0–3 kcal/mol. Complete triplicate data are available in Figure S1a–d in the Supplementary Materials.

The NMR-based Protein Data Bank (PDB) [40,41] entry for the heparin dodecasaccharide [-4IdoA2S*α*1–4GlcNS6S*α*1-]_6_ (PDB ID 1HPN [42]) consists of two structure models. In one model (“MODEL 2”), all IdoA2S residues have the ^1^C_4_ pucker conformation and all IdoA2S*α*1–4GlcNS6S linkages have (*φ*, *ψ*) = (–77°, 133°). In the other model (“MODEL 1”), all IdoA2S residues have the ^2^S_O_ pucker conformation and all IdoA2S*α*1–4GlcNS6S linkages have (*φ*, *ψ*) = (–55°, 135°). These differences in (*φ*, *ψ*) values between the two models are recapitulated in the **HS1*n*** disaccharide data here, wherein these disaccharides have their global-minimum Δ*G*(*φ*, *ψ*) in the neighborhood of (*φ*, *ψ*) = (−90°, 135°) for the ^1^C_4_ pucker state, with a shift to *φ* = −60° for the ^2^S_O_ pucker state (Figure 4; see Table S2 for exact values). For the dodecasaccharide-corresponding **HS13** disaccharide IdoA2S*α*1–4GlcNS6S*α*1-*O*-Me in particular, the data in Figure 4 are Δ*G*(−77.5°, 132.5°) = +0.3 kcal/mol for the ^1^C_4_ pucker state and Δ*G*(−55.0°, 135.0°) = +0.8 kcal/mol for the ^2^S_O_ pucker state. The global minimum in all cases in the present work is situated in a broad basin, and increasing sulfation and having IdoA[2S] in a boat-like state (i.e., either ^2^S_O_ or B_3,O_) tend to cause a reduction in the extent of the basin or the emergence of two or three distinct minima in that region of (*φ*, *ψ*) space. For example, **HS11** with a ^1^C_4_ IdoA pucker has a basin with its lowest Δ*G* region spanning 60° < *ψ* < 150°, whereas with a ^2^S_O_ IdoA pucker, this span shrinks to 105° < *ψ* < 150° and a distinct nearby local minimum, Δ*G*(−120°, 90°) = +2 kcal/mol, emerges.

**Figure 5:**
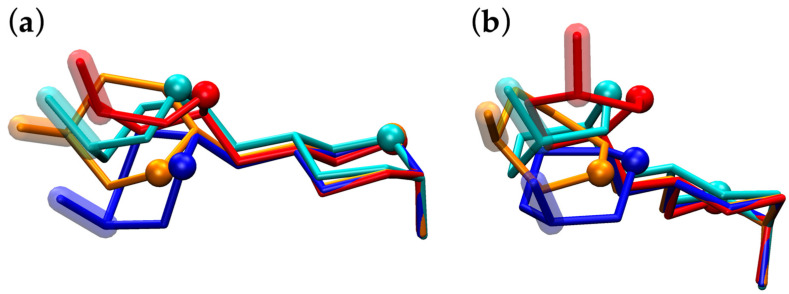
**HS11** simulation snapshots from global-minimum Δ*G*(*φ*, *ψ*) basins. Snapshots have been aligned using the GlcNS C1, O1, C2, and O5 atoms, and are shown from two perspectives (**a**,**b**), with GlcNS located on the right in both perspectives, O5 atoms represented as spheres, and IdoA C4–O4 bonds highlighted. Snapshots from a simulation where IdoA was restrained to the ^1^C_4_ pucker are colored cyan [(*φ*, *ψ*) = (−79.7°, 127.8°)] and blue [(*φ*, *ψ*) = (−85.1°, 90.5°)] and those where IdoA was restrained to ^2^S_O_ are colored orange [(*φ*, *ψ*) = (−121.8°, 89.7°)] and red [(*φ*, *ψ*) = (−66.8°, 126.7°)]. Snapshots were selected based on their (*φ*, *ψ*) proximities to local minima within the global-minimum Δ*G*(*φ*, *ψ*) basins located at *ψ* > 0° (Figure 4: **HS11** ^1^C_4_ and ^2^S_O_).

The molecular graphics of **HS11** simulation snapshots with (*φ*, *ψ*) values closest to the exact locations of the minima in this upper-left region of (*φ*, *ψ*) space demonstrate the qualitative differences in their overall conformations. GAGs are attached through their reducing ends to the protein core of PGs; therefore, taking disaccharide conformations from these Δ*G*(*φ*, *ψ*) basins and aligning them using the reducing end atoms of the GlcNS moiety provides perspective on how the GAG biopolymer orientations will deviate as they extend away from the reducing end. This is illustrated in Figure 5, which shows that the interplay between pucker state and preferred (*φ*, *ψ*) leads to a ^1^C_4_-containing conformation (Figure 5, cyan) being more structurally similar in this regard to the two ^2^S_O_-containing conformations (Figure 5, orange and red) than to the other ^1^C_4_-containing conformation (Figure 5, blue). Additionally, this first ^1^C_4_-containing conformation is more structurally similar to either of the two ^2^S_O_-containing conformations than the two ^2^S_O_-containing conformations are to each other.

The adjacent minima in the ^2^S_O_ global-minimum Δ*G*(*φ*, *ψ*) basin become more distinct as the free-energy barrier for escape from the global minimum to the nearby local minimum increases from 3 kcal/mol in **HS11** (Figure 4: **HS11** ^2^S_O_) to 6 kcal/mol in **HS14** (Figure 4: **HS14** ^2^S_O_), which demonstrates the effects of disaccharide sulfation in addition to IdoA pucker on Δ*G*(*φ*, *ψ*).

**Figure 6:**
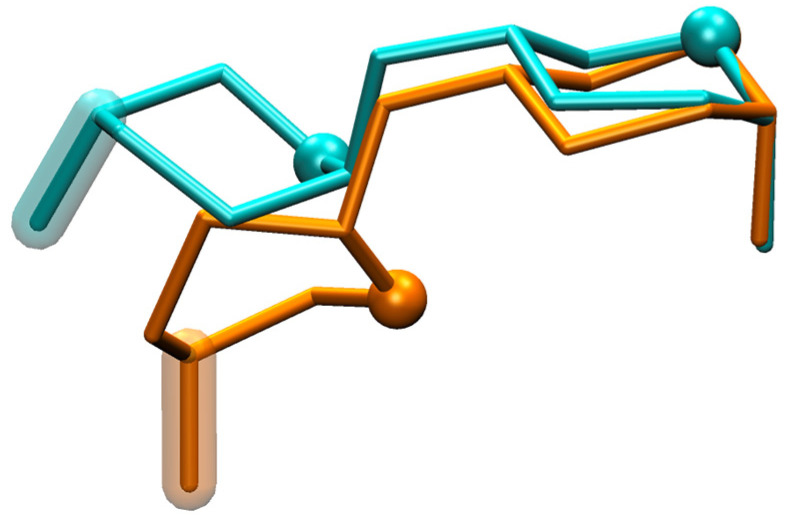
**HS14** simulation snapshots from secondary-minimum Δ*G*(*φ*, *ψ*) basins. Snapshots have been aligned using the GlcNS3S6S C1, O1, C2, and O5 atoms, with GlcNS3S6S located on the right, O5 atoms represented as spheres, and IdoA C4–O4 bonds highlighted. The snapshot from a simulation where IdoA was restrained to the ^1^C_4_ pucker is colored cyan [(*φ*, *ψ*) = (−96.5°, −82.5°)] and where IdoA was restrained to ^2^S_O_ is colored orange [(*φ*, *ψ*) = (−63.4°, −48.6°)]. Snapshots were selected based on their (*φ*, *ψ*) proximities to the local minima within the secondary-minimum Δ*G*(*φ*, *ψ*) basins. These minima, located at *ψ* < 0°, have Δ*G*(*φ*, *ψ*) = +1.3 kcal/mol for both the ^1^C_4_ and ^2^S_O_ pucker states (Figure 4: **HS14** ^1^C_4_ and ^2^S_O_).

**HS1*n*** disaccharides have a secondary-minimum basin near (−90°, −60°) that is distinct from the global-minimum basin (Figure 4). As with the global-minimum basin, this secondary-minimum basin changes as a function of both sulfation and IdoA pucker. This is most dramatically illustrated for the highly sulfated disaccharide **HS14**, which contains 3,6-di-*O*-sulfated GlcNS (GlcNS3S6S) as well as IdoA2S, where the basin has a minimum centered at (−97.5°, −80.0°) for the ^1^C_4_ pucker and at (−60.0°, −47.5°) for the ^2^S_O_ pucker. Again, the molecular graphics of simulation snapshots with (*φ*, *ψ*) values closest to the exact locations of these minima show the qualitative differences between the favored conformations for the ^1^C_4_ and ^2^S_O_ forms (Figure 6).

**Figure 7:**
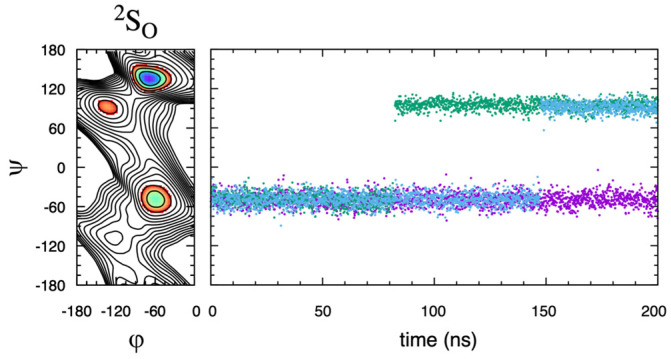
**HS14** kinetic trapping in a metastable state. Δ*G*(*φ*, *ψ*) from a 200 ns eABF simulation (**left frame**) compared to the time series for *ψ* from triplicate 200 ns nonbiased simulations (**right frame**). IdoA was restrained to ^2^S_O_ in both eABF and nonbiased simulations.

Based on the Δ*G*(*φ*, *ψ*) data here, this secondary-minimum basin can behave as a metastable state. For **HS14**, the barrier to escape from this secondary-minimum basin to the global-minimum basin is 4 kcal/mol when IdoA2S is in the ^1^C_4_ pucker state and 6 kcal/mol when it is in the ^2^S_O_ pucker state. Repeating the ^2^S_O_-restrained **HS14** triplicate simulations but with standard (nonbiased) molecular dynamics instead of eABF sampling and with the starting conformation in the secondary-minimum basin at (*φ*, *ψ*) = (−60.0°, −47.5°) demonstrates the kinetic trapping associated with a metastable state. Two of the trajectories make a single transition from the initial metastable state to the local minimum at (−132.5°, 92.5°) and the third trajectory remains in the starting metastable state for the entire 200 ns (Figure 7). That is to say, none of the three 200 ns nonbiased molecular dynamics trajectories sample the global minimum, and the lack of transitions implies a nonbiased sampling requirement beyond the 1-microsecond timescale. Consequently, care should be taken to ensure that the (*φ*, *ψ*) degrees-of-freedom are appropriately sampled when attempting to quantitate the conformational properties of GAG polymers containing IdoA*α*1–4GlcNS-type subunits. It is important to acknowledge that interactions beyond nearest-neighbor residues, which are not possible in disaccharides but can occur in GAG polymers, may increase the free energy of a metastable state and render it less thermodynamically relevant. With the aim of addressing this question, the eABF protocol was applied to the **HS14**-type IdoA2S*α*1–4GlcNS3S6S linkage in the GlcNS3S6S*α*1–4IdoA2S*α*1–4GlcNS3S6S*α*1-*O*-Me trisaccharide. While the overall appearance of the free-energy surface Δ*G*(*φ*, *ψ*) was the same as in the disaccharide, a firm conclusion could not be reached owing to poor convergence among the triplicate data (Figure S2).

**Figure 8:**
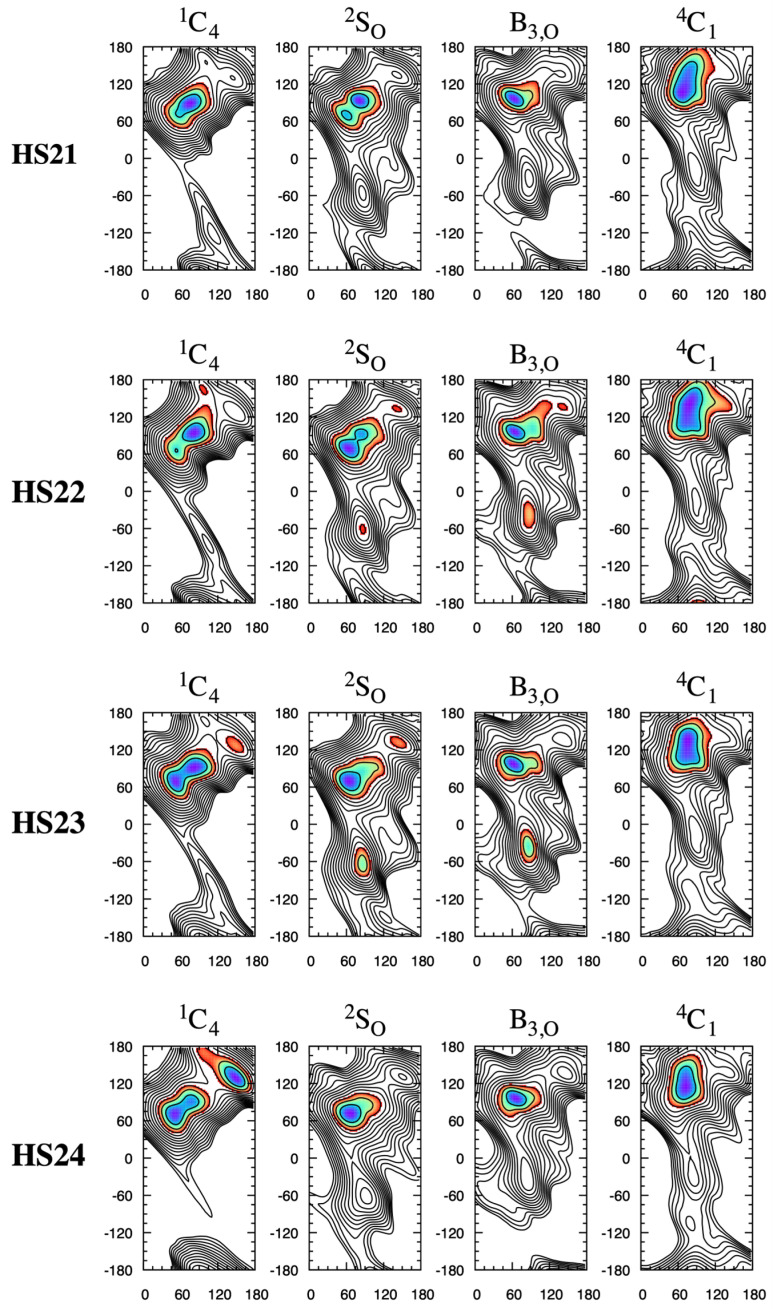
**HS2*n*** Δ*G*(*φ*, *ψ*) as a function of IdoA pucker. Each row is for a single disaccharide and each column is for a single IdoA pucker state. *φ* is on the *x*-axis and *ψ* is on the *y*-axis, with their values in degrees. Δ*G*(*φ*, *ψ*) is in kcal/mol with contours every 1 kcal/mol from 0 to 15 kcal/mol and is colored from blue to red in the range 0–3 kcal/mol. Complete triplicate data are available in Figure S1e–h in the Supplementary Materials.

**Figure 9:**
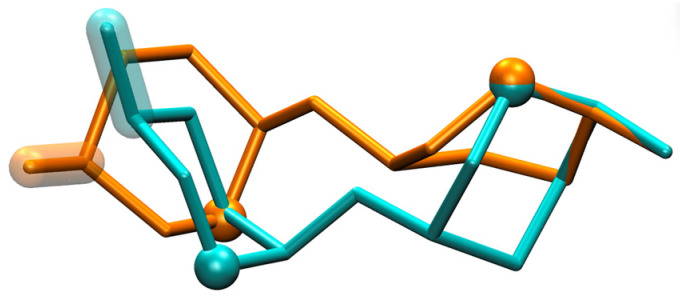
**HS22** simulation snapshots from global-minimum Δ*G*(*φ*, *ψ*) basins. Snapshots have been aligned using the IdoA2S C1, O1, C2, and O5 atoms, with IdoA2S located on the right, O5 atoms represented as spheres, and GlcNS C4–O4 bonds highlighted. The snapshot from a simulation where IdoA was restrained to the ^1^C_4_ pucker is colored cyan [(*φ*, *ψ*) = (82.7°, 95.6°)] and where IdoA was restrained to ^2^S_O_ is colored orange [(*φ*, *ψ*) = (59.6°, 68.9°)]. Snapshots were selected based on their (*φ*, *ψ*) proximities to the absolute minima within the global-minimum Δ*G*(*φ*, *ψ*) basins located at *ψ* > 0° (Figure 8: **HS22** ^1^C_4_ and ^2^S_O_).

**HS2*n*** disaccharides show similar behavior with regard to changes in the shape of the global-minimum Δ*G*(*φ*, *ψ*) basin as a function of the ^1^C_4_ vs. ^2^S_O_ pucker state. For example, for **HS22**, which is the GlcNS*α*1–4IdoA2S*α*1-*O*-Me disaccharide, the most favorable conformation within the basin is (*φ*, *ψ*) = (82.5°, 95.0°) when the pucker state is ^1^C_4_ and (*φ*, *ψ*) = (62.5°, 70.0°) when the pucker state is ^2^S_O_ (Figure 8). As with the **HS1*n*** disaccharides, the change in the preferred (*φ*, *ψ*) as a function of pucker state for a given sulfoform leads to a qualitative change in the direction along which a GAG would extend from the non-reducing end of the disaccharide (Figure 9).

The IdoA*α*1–4GlcNS and GlcNS*α*1–4IdoA glycosidic linkages in heparin have been noted to be flexible and rigid, respectively [33], which is captured in the present Δ*G*(*φ*, *ψ*) data. Specifically, **HS1*n*** Δ*G*(*φ*, *ψ*) data, which relate to IdoA*α*1–4GlcNS-type linkages, have two distinct stable basins for both ^1^C_4_ and ^2^S_O_ pucker states. For these **HS1*n*** disaccharides, a global-minimum basin is located at *ψ* > 0° and a secondary-minimum basin is located at *ψ* < 0°, and Δ*G*(*φ*, *ψ*) = +0.5–2 kcal/mol for the local minimum in this latter basin, depending upon the specific sulfoform (Figure 4). In contrast, **HS2*n*** Δ*G*(*φ*, *ψ*) data, which relate to GlcNS*α*1–4IdoA-type linkages, lack low (stable) free-energy secondary-minimum basins (Figure 8), which is consistent with less overall glycosidic linkage flexibility.

Sattelle et al., in analyzing their microsecond-timescale all-atom explicit-solvent molecular dynamics results on various heparin/HS oligosaccharides in the context of available experimental data, note that solution NMR spectroscopy shows overlapping (*φ*, *ψ*) distributions when 2-*O*-sulfo-IdoA (IdoA2S) is in the ^1^C_4_ vs. ^2^S_O_ pucker state [42,43,44]. Haasnoot et al. conclude their recent comprehensive study of idopyranose ring puckering by asserting that glycosidic linkages in heparin are relatively stiff [16] and referencing an earlier review from Mulloy and Forster [45]. Primary research from Mulloy et al. states that “The iduronate residues in [heparin polysaccharide] sequences may adopt either the ^1^C_4_ chair or the twist-boat conformations without causing major changes to the conformations of the glycosidic linkages,” based on a combination of NMR spectroscopy and molecular modeling [46]. Minimum-energy molecular models from Ferro et al. showed that “in heparin sequences conversion of one [IdoA2S] residue from conformation ^1^C_4_ to ^2^S_O_ can occur…without substantially affecting the position of the distant residues,” and with little differences in (*φ*, *ψ*) values [47]. Subsequent NMR NOE [48,49,50] and RDC [51] studies supplemented with molecular modeling have additionally been used to support the view that “the chair-skew boat equilibrium of IdoA has only a limited effect on the glycosidic linkage conformation of heparin [51].”

The results from the present work show that, while the IdoA[2S] pucker state in heparin/HS disaccharides does not alter the general (*φ*, *ψ*) location of the global-minimum Δ*G*, pucker states do lead to qualitative changes in the shapes and sizes of the global-minimum Δ*G*(*φ*, *ψ*) basins, including their splitting into distinct minima in some cases. As discussed above, these present results are in good agreement with the NMR-based PDB entry for the heparin dodecasaccharide [-4IdoA2S*α*1–4GlcNS6S*α*1-]_6_ with regard to changes in **HS1*n***-type IdoA2S*α*1–4GlcNS6S linkage (*φ*, *ψ*) correlating with the switch from the ^1^C_4_ pucker state to the ^2^S_O_ pucker state. With regard to the **HS2*n***-type GlcNS6S*α*1–4IdoA2S linkages in the PDB entry, the model with its IdoA2S residues in the ^1^C_4_ pucker conformation has its GlcNS6S*α*1–4IdoA2S linkages at (*φ*, *ψ*) = (79°, 88°). The other model, which has its IdoA2S residues in the ^2^S_O_ pucker conformation, has its GlcNS6S*α*1–4IdoA2S linkages at (*φ*, *ψ*) = (108°, 83°). Unlike for **HS1*n***-type linkages, the shifts seen in the **HS2*n*** disaccharide data are not consistent with those in the NMR structure. On the one hand, the corresponding disaccharide sulfoform **HS23** is in good agreement for the ^1^C_4_ pucker state, with Δ*G*(80.0, 87.5) = +0.1 kcal/mol, as shown in Figure 8. On the other hand, **HS23** with the ^2^S_O_ pucker state has Δ*G*(110.0, 82.5) = +2.5 kcal/mol, as shown in Figure 8, which is in rather poor agreement with the corresponding NMR structure. As the conditions for the disaccharide simulations mimic those used in NMR structure determination, it remains to be determined whether the apparent disagreement is a result of the polymer context and interactions beyond nearest-neighbor residues or whether it is a manifestation of force field inaccuracy. A future test of the force field might entail 10-microsecond or longer nonbiased simulations on the heparin dodecasaccharide [-4IdoA2S*α*1–4GlcNS6S*α*1-]_6_ to enable converged sampling of the glycosidic linkage and IdoA2S pucker degrees-of-freedom, with subsequent computation of NMR NOE values from the generated molecular dynamics ensemble for direct comparison with the experimental data used to generate the two NMR structure models. This will, of course, require that the force field be able to accurately represent the thermodynamic equilibrium between the IdoA2S ^1^C_4_ and ^2^S_O_ pucker states in a nonbiased simulation of this heparin polymer, which remains to be determined.

**Figure 10:**
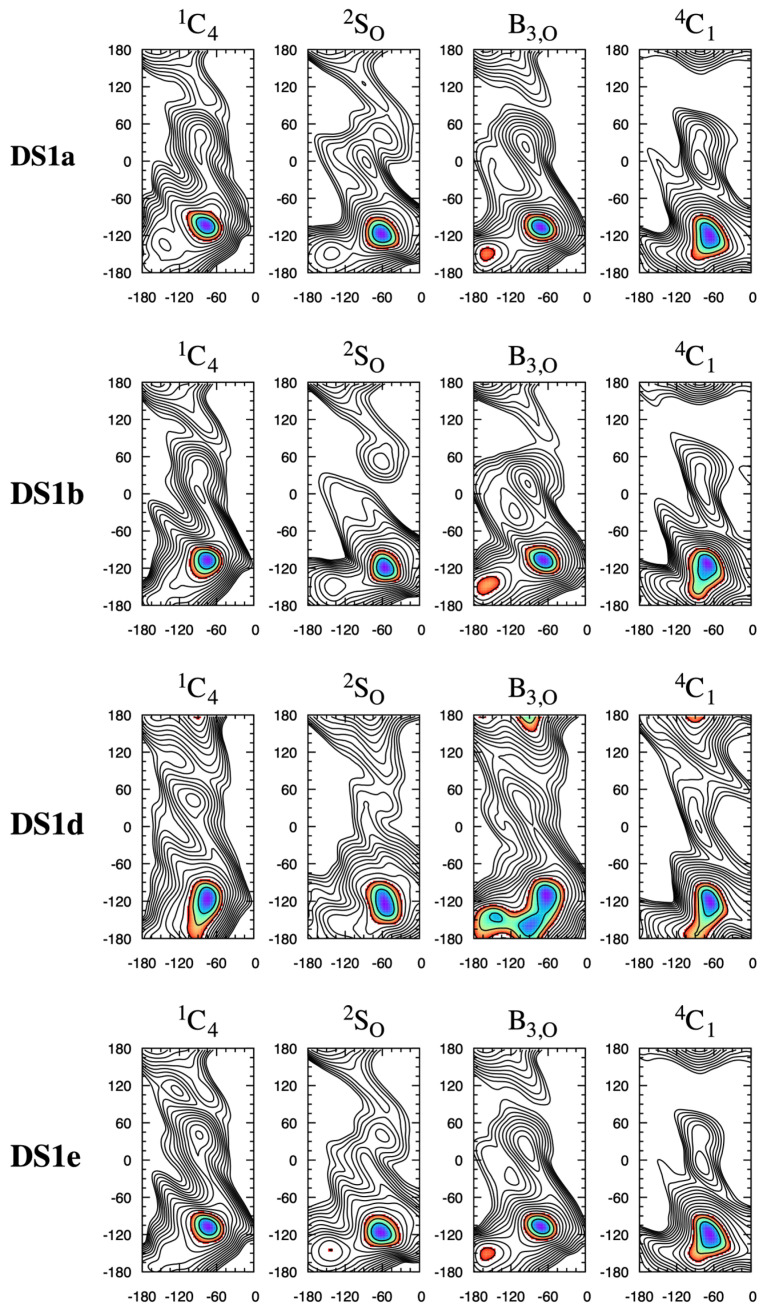
**DS1*x*** Δ*G*(*φ*, *ψ*) as a function of IdoA pucker. Each row is for a single disaccharide and each column is for a single IdoA pucker state. *φ* is on the *x*-axis and *ψ* is on the *y*-axis, with their values in degrees. Δ*G*(*φ*, *ψ*) is in kcal/mol with contours every 1 kcal/mol from 0 to 15 kcal/mol and is colored from blue to red in the range 0–3 kcal/mol. Complete triplicate data are available in Figure S1i–l in the Supplementary Materials.

**Figure 11:**
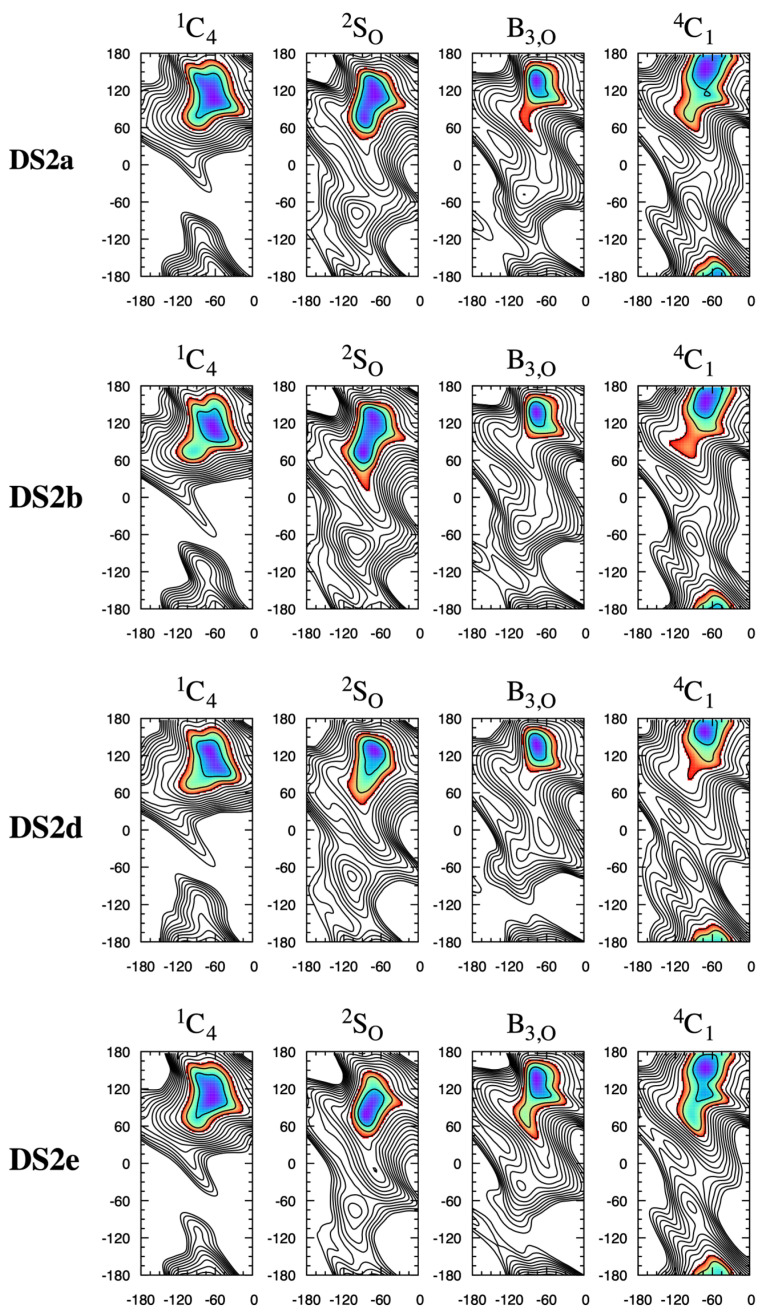
**DS2*x*** Δ*G*(*φ*, *ψ*) as a function of IdoA pucker. Each row is for a single disaccharide and each column is for a single IdoA pucker state. *φ* is on the *x*-axis and *ψ* is on the *y*-axis, with their values in degrees. Δ*G*(*φ*, *ψ*) is in kcal/mol with contours every 1 kcal/mol from 0 to 15 kcal/mol and is colored from blue to red in the range 0–3 kcal/mol. Complete triplicate data are available in Figure S1m–p in the Supplementary Materials.

**Figure 12:**
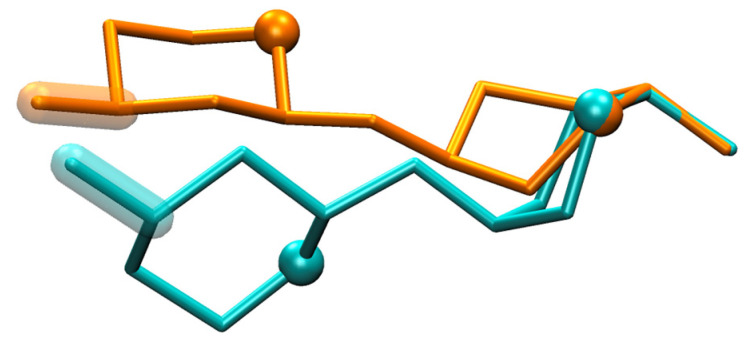
**DS2b** simulation snapshots from global-minimum Δ*G*(*φ*, *ψ*) basins. Snapshots have been aligned using the IdoA2S C1, O1, C2, and O5 atoms, with IdoA2S located on the right, O5 atoms represented as spheres, and GalNAc4S C3–O3 bonds highlighted. The snapshot from simulation where IdoA was restrained to the ^1^C_4_ pucker is colored cyan [(*φ*, *ψ*) = (−62.3°, 113.7°)] and where IdoA was restrained to ^2^S_O_ is colored orange [(*φ*, *ψ*) = (−85.3°, 74.0°)]. Snapshots were selected based on their (*φ*, *ψ*) proximities to the absolute minima within the global-minimum Δ*G*(*φ*, *ψ*) basins located at *ψ* > 0° (Figure 11: **DS2b** ^1^C_4_ and ^2^S_O_).

Compared to **HS1*n*** and **HS2*n*** disaccharides, **DS1*x*** disaccharides undergo very little change in global-minimum Δ*G*(*φ*, *ψ*) basins as a function of ^1^C_4_ vs. ^2^S_O_ pucker state (Figure 10). This is likely because the basin itself is of relatively small extent in both the *φ* and *ψ* directions compared to those for the **HS** disaccharides. In contrast to **DS1*x*** disaccharides, **DS2*x*** disaccharide global-minimum Δ*G*(*φ*, *ψ*) basins are subject to pucker state-dependent changes, which is not surprising since those basins are of substantially larger extent in both the *φ* and *ψ* directions (Figure 11). For example, **DS2b** global-minimum Δ*G*(*φ*, *ψ*) structures from ^1^C_4_ vs. ^2^S_O_ pucker state simulations have qualitatively different GalNAc 4-sulfate (GalNAc4S) O3 atom positions and C3–O3 bond vectors (Figure 12). The differences in basin extents are consistent with prior combined NMR and molecular modeling analysis of **DS11**- and **DS21**-type glycosidic linkages in a DS-derived tetrasaccharide, as are the (*φ*, *ψ*) values of the global-minimum Δ*G*(*φ*, *ψ*) structures [52] (*n.b.*: the referenced study used hydrogen atom (*φ*, *ψ*) definitions; comparison was performed using values of hydrogen atom-defined (*φ*, *ψ*) taken directly from simulation snapshots from the present work instead of IUPAC definitions as used throughout the rest of the present work). The locations of the basins in Figure 10 and Figure 11 are also consistent with 10-microsecond all-atom explicit-solvent nonbiased molecular dynamics simulations of DS decasaccharides [53]. Unlike in the case of heparin above, there is no solution structure of DS available in the PDB (PDB Advanced Search Query Builder: “Full Text: dermatan” AND “Structure Attributes: Experimental Method (Broader Categories) is NMR”; search performed 18 November 2025).

While existing experimental data speak to the thermodynamic primacy of the ^1^C_4_ and ^2^S_O_ IdoA pucker states in heparin/HS and DS biopolymers [16,42,47,50,51,52,54,55,56], the present results show that, in those instances when IdoA takes on a B_3,O_ or ^4^C_1_ pucker state [16], a substantial change can also occur in the preferred conformations of the glycosidic linkages. For the B_3,O_ pucker state, this is especially true for the glycosidic linkages in the **HS13** and **HS14** disaccharides (Figure 4) and in the **DS1d** disaccharides (Figure 10). For the ^4^C_1_ pucker state, this is most evident in **HS2*n*** disaccharides (Figure 8) and **DS2*x*** disaccharides (Figure 11). That said, it is important to keep in mind that the B_3,O_ and ^4^C_1_ pucker states do not meaningfully contribute to the puckering equilibrium of O1,O4-disubstituted IdoA[2S], as in GAGs.

## 3. Conclusions

The present study puts forth a comprehensive collection of all-atom explicit-solvent molecular dynamics data demonstrating that the GAG disaccharide IdoA pucker and sulfoform can both affect Δ*G*(*φ*, *ψ*). The overall picture that emerges is that IdoA shifting between its thermodynamically important ^1^C_4_ and ^2^S_O_ pucker states in heparin/HS disaccharides can lead to qualitative changes in the overall conformation of the disaccharides not only directly through pucker geometry but also through pucker-dependent changes in the broad global-minimum Δ*G*(*φ*, *ψ*) basins for IdoA*α*1–4GlcNS-type and GlcNS*α*1–4IdoA-type glycosidic linkages. For DS, this effect is less dramatic, as ^1^C_4_ vs. ^2^S_O_ pucker-dependent change is limited to the basins for only GalNAc*β*1–4IdoA-type linkages. Across the considered heparin/HS and DS disaccharides, Δ*G*(*φ*, *ψ*) may also change as a function of sulfoform, such that both IdoA pucker and the GAG sulfoform should be considered contributing factors to preferred glycosidic linkage (*φ*, *ψ*) values.

The IdoA*α*1–4GlcNS-type Δ*G*(*φ*, *ψ*) data show the presence of a prominent secondary-minimum basin in a distinct quadrant of (*φ*, *ψ*) space. Especially in the context of the most highly sulfated variant of this disaccharide considered here, this secondary-minimum basin may act as a metastable state in 1-microsecond-timescale molecular dynamics simulations. Therefore, caution should be taken to ensure the system is not trapped in this metastable (*φ*, *ψ*) state when attempting, for example, to quantitate free-energy differences between ^1^C_4_ and ^2^S_O_ pucker states in sub-microsecond all-atom explicit-solvent molecular dynamics simulations in which the pucker transition is accelerated with a biased sampling method but (*φ*, *ψ*) sampling is nonbiased.

While it is tempting to construct GAG polymers for structural comparison using the (*φ*, *ψ*) values associated with disaccharide Δ*G*(*φ*, *ψ*) = 0 kcal/mol (Table S2), doing so will produce non-physical “virtual conformations” because, in reality, these polymers are highly flexible [57]. A more sophisticated approach might be to apply a Metropolis Monte Carlo scheme to select (*φ*, *ψ*) values using Boltzmann probabilities computed from Δ*G*(*φ*, *ψ*) [58]. With regard to the present data, such an approach may not be able to capture perturbations to disaccharide Δ*G*(*φ*, *ψ*) arising from the polymer context. Such perturbations may arise from direct interactions between non-adjacent residues in the polymer sequence. They may also arise from water-mediated or ion-mediated interactions, or from hydrophobic forces. As such, the present disaccharide Δ*G*(*φ*, *ψ*) data can serve as a point of reference for analysis of GAG polymer simulations. If (*φ*, *ψ*) distributions from a GAG polymer simulation deviate substantially from the Δ*G*(*φ*, *ψ*) for the constituent disaccharides, one possibility is that there is incomplete sampling in the GAG polymer simulation. Another is that the polymer context induces perturbations to disaccharide Δ*G*(*φ*, *ψ*). This type of comparative analysis may be useful when using the results of all-atom explicit-solvent GAG simulations as a basis for the direct construction of conformations of longer GAG polymers [59,60] or for the development of coarse-grained GAG force fields [43,53,61,62,63,64].

## 4. Methods

Four initial conformations were constructed for each disaccharide, with the *α*IdoA moiety being in the ^1^C_4_, ^2^S_O_, B_3,O_, or ^4^C_1_ pucker state. Coordinates for these pucker states were extracted from prior triplicate 200 ns eABF [29,30] pucker sampling molecular dynamics simulations of *α*IdoA, as described in reference [17]. The dataset from those prior simulation trajectories comprised 60,000 snapshots. For each of the four pucker states, the most “ideal” pucker geometry, as determined by CP (*θ*, *ϕ*) [8], was extracted from the 60,000-snapshot dataset. Ideal pucker geometries were defined as follows:

^1^C_4_: *θ*_ideal_ ≡ 180°;
^2^S_O_: (*θ*_ideal_, *ϕ*_ideal_) ≡ (90°, 150°);
B_3,O_: (*θ*_ideal_, *ϕ*_ideal_) ≡ (90°, 180°);
^4^C_1_: *θ*_ideal_ ≡ 0°.

The closeness-to-idealness of a conformation was defined as (*θ* − *θ*_ideal_)^2^ for ^1^C_4_ and ^4^C_1_ and as (*θ* − *θ*_ideal_)^2^ + (*ϕ* − *ϕ*_ideal_)^2^ for ^2^S_O_ and ^4^C_1_. (*θ*, *ϕ*) for each snapshot was computed with the CHARMM program v. 49b2 [65,66]. Table 2 lists (*θ*, *ϕ*) for each of the four extracted pucker state coordinates found to be closest to idealness in the dataset and deemed “(*θ*_0_, *ϕ*_0_),” along with the values of the ring dihedrals *Φ*_0,*i*_ for those coordinates.

The CHARMM program was used for all subsequent system construction. Missing disaccharide coordinates were added to *α*IdoA coordinates based on force field topology internal coordinates [24,25,26,27,28]. Internal coordinates were from the “jul24” release of the CHARMM36 force field available as “toppar_c36_jul24.tgz” from https://mackerell.umaryland.edu/charmm_ff.shtml (accessed on 14 March 2025) with minor custom modifications to facilitate structure building. These custom modifications are available in the form of a diff file in the Supplementary Materials to be applied as a patch to the original “top_all36_carb.rtf” file included in “toppar_c36_jul24.tgz.” Pucker states for GlcNS, GalNAc, and their derivatives were ^4^C_1_ per force field topology internal coordinates.

Each disaccharide was solvated in a cube of CHARMM-modified TIP3P water molecules [67,68] with an edge length of 30 Å plus the dimension of the disaccharide along the *x*-axis after alignment of its principal geometric axis with that axis, and with the density of water molecules in the cube being the experimental density of liquid water. Water molecules within 3 Å of the disaccharide were deleted, and neutralizing sodium ions [69,70] were added by randomly replacing water molecules located at least 6 Å from the disaccharide.

Molecular dynamics simulations on each system were performed in triplicate with NAMD software v. 3.0.1 [71] (NAMD was developed by the Theoretical and Computational Biophysics Group in the Beckman Institute for Advanced Science and Technology at the University of Illinois at Urbana-Champaign), and using the internally consistent CHARMM36 additive (nonpolarizable) carbohydrate, water, and ion force field parametrizations referenced above. While each member of the triplicate was started from identical initial disaccharide/water/ion coordinates, the triplicates evolved as different trajectories due to randomness in the velocity during the heating stage and from the use of Langevin thermostating and barostating (see below). Systems were simulated under periodic boundary conditions [72] with a 10 Å spherical cutoff. Lennard–Jones (LJ) interactions were smoothly switched off over the 8–10 Å interval [73] and LJ interactions beyond the cutoff distance were accounted for with an isotropic correction [74]. Electrostatic interactions beyond the cutoff distance were accounted for with the particle mesh Ewald (PME) method [75] with a 1 Å grid spacing.

After minimization to remove bad contacts, each system was heated in triplicate by assigning random velocities from a distribution appropriate for the target temperature of 298 K, and performing this same type of velocity assignment every 1000 integration steps for a total of 20,000 steps. Bonds to hydrogen atoms as well as the internal geometry of water molecules were constrained to their force field equilibrium parameter values using the SHAKE [76] and SETTLE algorithms [77]; disaccharide conformations were maintained with harmonic restraints on non-hydrogen disaccharide atom Cartesian coordinates, an average pressure of 1 atm was targeted with an isotropic Nosé–Hoover Langevin piston [78,79], and the equations of motion were integrated using a 0.5 fs timestep with the velocity Verlet integrator [80].

A 200 ns eABF molecular dynamics simulation was started using the final positions and velocities of each heating run. These followed the same protocol for heating except that Cartesian coordinate restraints were replaced with an internal geometry-based ring restraining potential on IdoA[2S] to maintain its starting pucker conformation (Table 2); replaced random velocity assignment was replaced with Langevin thermostating at 298 K; the timestep was 2 fs; and eABF was used to enhance sampling on (*φ*, *ψ*). The Colvars module [81] within NAMD was used for the ring restraining potential $\frac{1}{2}k\sum_{i=1}^{6}{\left(\frac{\Phi_{i}-\Phi_{0,i}}{w}\right)}^{2}$, where the ring dihedrals *Φ* are defined in Table 2, *w* = 5°, and *k* = 1 kcal/mol. IdoA[2S] CP (*θ*, *ϕ*) distributions were analyzed for each of the 192 200 ns eABF molecular dynamics trajectories to confirm that the ring restraining potential properly kept the pucker conformation in the desired ^1^C_4_, ^2^S_O_, B_3,O_, or ^4^C_1_ pucker state. Figure S3 in the Supplementary Materials shows these data from four **HS11** simulations, each with IdoA restrained to the ^1^C_4_, ^2^S_O_, B_3,O_, or ^4^C_1_ pucker state. Figure S3 demonstrates the effectiveness of the restraining potential in retaining the desired pucker state while allowing for moderate ring deformations that might lessen the strain on glycosidic dihedrals relative to combining very rigid ring geometries with eABF sampling. Data from the other 188 simulations are essentially the same as those in Figure S3. The Colvars module within NAMD was additionally used for eABF sampling [29,82]; CZAR estimation of free-energy gradients [29]; and Poisson integration for the free-energy surface [83]. The CHARMM program was used for post-simulation analysis of trajectory snapshots. Molecular graphics were created with VMD software v. 1.9.4a57 [84].

## Figures and Tables

**Table 1:** Sulfation patterns for heparin/HS and DS disaccharides in the present work.

Disaccharide Template (Sequence *)	Sulfation Pattern	–O*R*_1_ **	–O*R*_2_ **	–O*R*_3_ **
**HS1*n*** (IdoA*α*1–4GlcNS*α*1-*O*-Me) **HS2*n*** (GlcNS*α*1–4IdoA*α*1-*O*-Me)	***n*** = **1**	–OH	–OH	–OH
	***n*** = **2**	–OH	–OH	–OSO_3_^−^
	***n*** = **3**	–OH	–OSO_3_^−^	–OSO_3_^−^
	***n*** = **4**	–OSO_3_^−^	–OSO_3_^−^	–OSO_3_^−^
**DS1*x*** (IdoA*α*1–3GalNAc*β*1-*O*-Me) **DS2*x*** (GalNAc*β*1–4IdoA*α*1-*O*-Me)	***x*** = **a**	–OSO_3_^−^	–OH	–OH
	***x*** = **b**	–OSO_3_^−^	–OH	–OSO_3_^−^
	***x*** = **d**	–OH	–OSO_3_^−^	–OSO_3_^−^
	***x*** = **e**	–OSO_3_^−^	–OSO_3_^−^	–OH

* IdoA: iduronate; GlcNS: *N*-sulfoglucosamine; GalNAc: *N*-acetylgalactosamine. ** Refer to Figure 3 for locations of –O*R* functional groups.

**Table 2:** CP parameter values (*θ*_0_, *ϕ*_0_) and ring dihedral values *Φ*_0,*i*_ * for *α*IdoA coordinates used to construct initial disaccharide conformations.

Pucker	*θ*_0_	*ϕ*_0_	*Φ*_0,1_	*Φ*_0,2_	*Φ*_0,3_	*Φ*_0,4_	*Φ*_0,5_	*Φ*_0,6_
^1^C_4_	179.72°	n/a	−61.4°	57.4°	−58.2°	65.5°	−65.1°	61.6°
^2^S_O_	89.92°	149.87°	27.7°	−56.4°	26.5°	29.5°	−60.1°	29.1°
B_3,O_	90.07°	180.03°	−2.6°	−53.9°	54.7°	0.6°	−60.8°	62.5°
^4^C_1_	0.17°	n/a	68.1°	−62.6°	66.2°	−70.6°	67.3°	−69.6°

* *i* = 1 defined by O5–C1–C2–C3; *i* = 2 by C1–C2–C3–C4; *i* = 3 by C2–C3–C4–C5; *i* = 4 by C3–C4–C5–O5; *i* = 5 by C4–C5–O5–C1; and *i* = 6 by C5–O5–C1–C2.

## Data Availability

The original contributions presented in this study are included in the article/Supplementary Materials. Further inquiries can be directed to the corresponding authors.

## Supplementary Materials

The following supporting information can be downloaded at https://www.mdpi.com/article/10.3390/molecules31030504/s1: Table S1: Abbreviations and chemical compositions for heparin/heparan sulfate and dermatan sulfate disaccharides; Table S2: Average (*φ*, *ψ*) for Δ*G*(*φ*, *ψ*) = 0 kcal/mol from triplicate eABF simulations as a function of IdoA[2S] pucker state; Table S3: Differences in (*φ*, *ψ*) for Δ*G*(*φ*, *ψ*) = 0 kcal/mol as a function of IdoA[2S] pucker state; Figure S1: Triplicate Δ*G*(*φ*, *ψ*) data as a function of IdoA pucker; Figure S2: Triplicate Δ*G*(*φ*, *ψ*) data for the IdoA2S*α*1–4GlcNS3S6S glycosidic linkage in the GlcNS3S6S*α*1–4IdoA2S*α*1–4GlcNS3S6S*α*1-*O*-Me trisaccharide; Figure S3: IdoA Cremer–Pople (*θ*, *ϕ*) distributions from 200 ns eABF molecular dynamics simulations; patch file “top_all36_carb.diff” to be applied to “top_all36_carb.rtf” from the “jul24” release of the CHARMM36 force field.

## Abbreviations

The following abbreviations are used in this manuscript:

3D	Three-dimensional
CP	Cremer–Pople
DS	Dermatan sulfate
eABF	Extended-system adaptive biasing force
GAG	Glycosaminoglycan
GalNAc	*N*-acetylgalactosamine
GalNAc4S	*N*-acetylgalactosamine 4-sulfate
Glc	Glucose
GlcNS	*N*-sulfoglucosamine
GlcNS3S6S	*N*-sulfoglucosamine 3,6-disulfate
HS	Heparan sulfate
IdoA	Iduronate
IdoA2S	Iduronate 2-sulfate
PDB	Protein Data Bank
PG	Proteoglycan

## References

[1] Merry C.L.R., Lindahl U., Couchman J., Esko J.D., Varki A., Cummings R.D., Esko J.D., Stanley P., Hart G.W., Aebi M., Mohnen D., Kinoshita T., Packer N.H., Prestegard J.H. (2022). Proteoglycans and Sulfated Glycosaminoglycans. Essentials of Glycobiology.

[2] Merry C.L.R. (2021). Exciting New Developments and Emerging Themes in Glycosaminoglycan Research. J. Histochem. Cytochem..

[3] Shi D., Sheng A., Chi L. (2021). Glycosaminoglycan-Protein Interactions and Their Roles in Human Disease. Front. Mol. Biosci..

[4] Perez S., Makshakova O., Angulo J., Bedini E., Bisio A., De Paz J.L., Fadda E., Guerrini M., Hricovini M., Hricovini M. (2023). Glycosaminoglycans: What Remains To Be Deciphered?. JACS Au.

[5] Ricard-Blum S., Vivès R.R., Schaefer L., Götte M., Merline R., Passi A., Heldin P., Magalhães A., Reis C.A., Skandalis S.S. (2024). A Biological Guide to Glycosaminoglycans: Current Perspectives and Pending Questions. FEBS J..

[6] Rao V.S.R., Qasba P.K., Balaji P.V., Chandrasekaran R. (2019). Conformation of Carbohydrates.

[7] Franconetti A., Gómez M., Ardá A., Poveda A., Jiménez-Barbero J. (2024). Elucidation of the Structure of Carbohydrates and Their Interactions by Nuclear Magnetic Resonance Spectroscopy. Translational Glycobiology in Human Health and Disease.

[8] Cremer D., Pople J.A. (1975). General Definition of Ring Puckering Coordinates. J. Am. Chem. Soc..

[9] IUPAC-IUB Joint Commission on Biochemical Nomenclature (JCBN) (1983). Symbols for Specifying the Conformation of Polysaccharide Chains. Recommendations 1981. Eur. J. Biochem..

[10] IUPAC-IUB Joint Commission on Biochemical Nomenclature (JCBN) (1980). Conformational Nomenclature for Five and Six-Membered Ring Forms of Monosaccharides and Their Derivatives: Recommendations 1980. Eur. J. Biochem..

[11] Casu B., Choay J., Ferro D.R., Gatti G., Jacquinet J.-C., Petitou M., Provasoli A., Ragazzi M., Sinay P., Torri G. (1986). Controversial Glycosaminoglycan Conformations. Nature.

[12] Casu B., Petitou M., Provasoli M., Sinaÿ P. (1988). Conformational Flexibility: A New Concept for Explaining Binding and Biological Properties of Iduronic Acid-Containing Glycosaminoglycans. Trends Biochem. Sci..

[13] Sattelle B.M., Hansen S.U., Gardiner J., Almond A. (2010). Free Energy Landscapes of Iduronic Acid and Related Monosaccharides. J. Am. Chem. Soc..

[14] Sattelle B.M., Almond A. (2011). Is N-Acetyl-D-Glucosamine a Rigid 4C1 Chair?. Glycobiology.

[15] Bose-Basu B., Zhang W., Kennedy J.L., Hadad M.J., Carmichael I., Serianni A.S. (2017). (13)C-Labeled Idohexopyranosyl Rings: Effects of Methyl Glycosidation and C6 Oxidation on Ring Conformational Equilibria. J. Org. Chem..

[16] Haasnoot C.A.G., de Gelder R., Kooijman H., Kellenbach E.R. (2020). The Conformation of the Idopyranose Ring Revisited: How Subtle O-Substituent Induced Changes Can Be Deduced from Vicinal 1H-NMR Coupling Constants. Carbohydr. Res..

[17] Guvench O., Martin D., Greene M. (2022). Pyranose Ring Puckering Thermodynamics for Glycan Monosaccharides Associated with Vertebrate Proteins. Int. J. Mol. Sci..

[18] Hogwood J., Mulloy B., Lever R., Gray E., Page C.P. (2023). Pharmacology of Heparin and Related Drugs: An Update. Pharmacol. Rev..

[19] Samsonov S.A., Pisabarro M.T. (2013). Importance of IdoA and IdoA(2S) Ring Conformations in Computational Studies of Glycosaminoglycan–Protein Interactions. Carbohydr. Res..

[20] Mulloy B., Khan S., Perkins S.J. (2011). Molecular Architecture of Heparin and Heparan Sulfate: Recent Developments in Solution Structural Studies. Pure Appl. Chem..

[21] Farrugia B.L., Melrose J. (2023). The Glycosaminoglycan Side Chains and Modular Core Proteins of Heparan Sulphate Proteoglycans and the Varied Ways They Provide Tissue Protection by Regulating Physiological Processes and Cellular Behaviour. Int. J. Mol. Sci..

[22] Chen C., Zhang X., Zhang W., Ding D., Loka R.S., Zhao K., Ling P., Wang S. (2025). Dermatan Sulfate: Structure, Biosynthesis, and Biological Roles. Biomolecules.

[23] Almond A. (2018). Multiscale Modeling of Glycosaminoglycan Structure and Dynamics: Current Methods and Challenges. Curr. Opin. Struct. Biol..

[24] Guvench O., Greene S.N., Kamath G., Brady J.W., Venable R.M., Pastor R.W., Mackerell A.D. (2008). Additive Empirical Force Field for Hexopyranose Monosaccharides. J. Comput. Chem..

[25] Guvench O., Hatcher E.R., Venable R.M., Pastor R.W., MacKerell A.D. (2009). CHARMM Additive All-Atom Force Field for Glycosidic Linkages between Hexopyranoses. J. Chem. Theory Comput..

[26] Guvench O., Mallajosyula S.S., Raman E.P., Hatcher E., Vanommeslaeghe K., Foster T.J., Jamison F.W., Mackerell A.D. (2011). CHARMM Additive All-Atom Force Field for Carbohydrate Derivatives and Its Utility in Polysaccharide and Carbohydrate-Protein Modeling. J. Chem. Theory Comput..

[27] Mallajosyula S.S., Guvench O., Hatcher E., MacKerell A.D. (2012). CHARMM Additive All-Atom Force Field for Phosphate and Sulfate Linked to Carbohydrates. J. Chem. Theory Comput..

[28] Yu W., He X., Vanommeslaeghe K., MacKerell A.D. (2012). Extension of the CHARMM General Force Field to Sulfonyl-Containing Compounds and Its Utility in Biomolecular Simulations. J. Comput. Chem..

[29] Lesage A., Lelièvre T., Stoltz G., Hénin J. (2017). Smoothed Biasing Forces Yield Unbiased Free Energies with the Extended-System Adaptive Biasing Force Method. J. Phys. Chem. B.

[30] Fu H., Shao X., Chipot C., Cai W. (2016). Extended Adaptive Biasing Force Algorithm. An On-the-Fly Implementation for Accurate Free-Energy Calculations. J. Chem. Theory Comput..

[31] Lutsyk V., Plazinski W. (2021). Conformational Properties of Glycosaminoglycan Disaccharides: A Molecular Dynamics Study. J. Phys. Chem. B.

[32] Plazinski W., Drach M. (2015). The Influence of the Hexopyranose Ring Geometry on the Conformation of Glycosidic Linkages Investigated Using Molecular Dynamics Simulations. Carbohydr. Res..

[33] Angulo J., Ardá A., Bertuzzi S., Canales A., Ereño-Orbea J., Gimeno A., Gomez-Redondo M., Muñoz-García J.C., Oquist P., Monaco S. (2024). NMR Investigations of Glycan Conformation, Dynamics, and Interactions. Prog. Nucl. Magn. Reson. Spectrosc..

[34] Marques C., Reis C.A., Vivès R.R., Magalhães A. (2021). Heparan Sulfate Biosynthesis and Sulfation Profiles as Modulators of Cancer Signalling and Progression. Front. Oncol..

[35] Wang W., Shi L., Qin Y., Li F. (2020). Research and Application of Chondroitin Sulfate/Dermatan Sulfate-Degrading Enzymes. Front. Cell Dev. Biol..

[36] Mizumoto S., Yamada S. (2022). The Specific Role of Dermatan Sulfate as an Instructive Glycosaminoglycan in Tissue Development. Int. J. Mol. Sci..

[37] Hayes A.J., Melrose J. (2021). Neural Tissue Homeostasis and Repair Is Regulated via CS and DS Proteoglycan Motifs. Front. Cell Dev. Biol..

[38] Malmström A., Bartolini B., Thelin M.A., Pacheco B., Maccarana M. (2012). Iduronic Acid in Chondroitin/Dermatan Sulfate: Biosynthesis and Biological Function. J. Histochem. Cytochem..

[39] Hsieh P.H., Thieker D.F., Guerrini M., Woods R.J., Liu J. (2016). Uncovering the Relationship between Sulphation Patterns and Conformation of Iduronic Acid in Heparan Sulphate. Sci. Rep..

[40] Berman H.M., Westbrook J., Feng Z., Gilliland G., Bhat T.N., Weissig H., Shindyalov I.N., Bourne P.E. (2000). The Protein Data Bank. Nucleic Acids Res..

[41] Burley S.K., Bhatt R., Bhikadiya C., Bi C., Biester A., Biswas P., Bittrich S., Blaumann S., Brown R., Chao H. (2025). Updated Resources for Exploring Experimentally-Determined PDB Structures and Computed Structure Models at the RCSB Protein Data Bank. Nucleic Acids Res..

[42] Mulloy B., Forster M.J., Jones C., Davies D.B. (1993). N.m.r. and Molecular-Modelling Studies of the Solution Conformation of Heparin. Biochem. J..

[43] Sattelle B.M., Shakeri J., Almond A. (2013). Does Microsecond Sugar Ring Flexing Encode 3D-Shape and Bioactivity in the Heparanome?. Biomacromolecules.

[44] Blundell C.D., Roberts I.S., Sheehan J.K., Almond A. (2009). Investigating the Molecular Basis for the Virulence of *Escherichia coli* K5 by Nuclear Magnetic Resonance Analysis of the Capsule Polysaccharide. Microb. Physiol..

[45] Mulloy B., Forster M.J. (2000). Conformation and Dynamics of Heparin and Heparan Sulfate. Glycobiology.

[46] Mulloy B., Forster M.J., Jones C., Drake A.F., Johnson E.A., Davies D.B. (1994). The Effect of Variation of Substitution on the Solution Conformation of Heparin: A Spectroscopic and Molecular Modelling Study. Carbohydr. Res..

[47] Ferro D.R., Provasoli A., Ragazzi M., Torri G., Casu B., Gatti G., Jacquinet J.C., Sinay P., Petitou M., Choay J. (1986). Evidence for Conformational Equilibrium of the Sulfated L-Iduronate Residue in Heparin and in Synthetic Heparin Mono- and Oligo-Saccharides: NMR and Force-Field Studies. J. Am. Chem. Soc..

[48] Mikhailov D., Linhardt J.R., Mayo H.K. (1997). NMR Solution Conformation of Heparin-Derived Hexasaccharide. Biochem. J..

[49] De Paz J.-L., Angulo J., Lassaletta J.-M., Nieto P.M., Redondo-Horcajo M., Lozano R.M., Giménez-Gallego G., Martín-Lomas M. (2001). The Activation of Fibroblast Growth Factors by Heparin: Synthesis, Structure, and Biological Activity of Heparin-Like Oligosaccharides. ChemBioChem.

[50] Zhang Z., McCallum S.A., Xie J., Nieto L., Corzana F., Jiménez-Barbero J., Chen M., Liu J., Linhardt R.J. (2008). Solution Structures of Chemoenzymatically Synthesized Heparin and Its Precursors. J. Am. Chem. Soc..

[51] Jin L., Hricovini M., Deakin J.A., Lyon M., Uhrin D. (2009). Residual Dipolar Coupling Investigation of a Heparin Tetrasaccharide Confirms the Limited Effect of Flexibility of the Iduronic Acid on the Molecular Shape of Heparin. Glycobiology.

[52] Silipo A., Zhang Z., Canada F.J., Molinaro A., Linhardt R.J., Jimenez-Barbero J. (2008). Conformational Analysis of a Dermatan Sulfate-Derived Tetrasaccharide by NMR, Molecular Modeling, and Residual Dipolar Couplings. ChemBioChem.

[53] Sattelle B.M., Shakeri J., Cliff M.J., Almond A. (2015). Proteoglycans and Their Heterogeneous Glycosaminoglycans at the Atomic Scale. Biomacromolecules.

[54] Forster M.J., Mulloy B. (1993). Molecular Dynamics Study of Iduronate Ring Conformation. Biopolymers.

[55] Van Boeckel C.A.A., Van Aelst S.F., Wagenaars G.N., Mellema J.-R., Paulsen H., Peters T., Pollex A., Sinnwell V. (1987). Conformational Analysis of Synthetic Heparin-like Oligosaccharides Containing α-L-idopyranosyluronic Acid. Recl. Trav. Chim. Pays-Bas.

[56] Ferro D.R., Provasoli A., Ragazzi M., Casu B., Torri G., Bossennec V., Perly B., Sinay P., Petitou M., Choay J. (1990). Conformer Populations of L-Iduronic Acid Residues in Glycosaminoglycan Sequences. Carbohydr. Res..

[57] Woods R.J. (2018). Predicting the Structures of Glycans, Glycoproteins, and Their Complexes. Chem. Rev..

[58] Lutsyk V., Wolski P., Plazinski W. (2024). The Conformation of Glycosidic Linkages According to Various Force Fields: Monte Carlo Modeling of Polysaccharides Based on Extrapolation of Short-Chain Properties. J. Chem. Theory Comput..

[59] Whitmore E.K., Vesenka G., Sihler H., Guvench O. (2020). Efficient Construction of Atomic-Resolution Models of Non-Sulfated Chondroitin Glycosaminoglycan Using Molecular Dynamics Data. Biomolecules.

[60] Whitmore E.K., Martin D., Guvench O. (2020). Constructing 3-Dimensional Atomic-Resolution Models of Nonsulfated Glycosaminoglycans with Arbitrary Lengths Using Conformations from Molecular Dynamics. Int. J. Mol. Sci..

[61] Bathe M., Rutledge G.C., Grodzinsky A.J., Tidor B. (2005). A Coarse-Grained Molecular Model for Glycosaminoglycans: Application to Chondroitin, Chondroitin Sulfate, and Hyaluronic Acid. Biophys. J..

[62] Samsonov S.A., Bichmann L., Pisabarro M.T. (2015). Coarse-Grained Model of Glycosaminoglycans. J. Chem. Inf. Model..

[63] Shivgan A.T., Marzinek J.K., Krah A., Matsudaira P., Verma C.S., Bond P.J. (2024). Coarse-Grained Model of Glycosaminoglycans for Biomolecular Simulations. J. Chem. Theory Comput..

[64] Lutsyk V., Plazinski W. (2025). Extending the Martini 3 Coarse-Grained Force Field to Hyaluronic Acid. J. Phys. Chem. B.

[65] Hwang W., Austin S.L., Blondel A., Boittier E.D., Boresch S., Buck M., Buckner J., Caflisch A., Chang H.T., Cheng X. (2024). CHARMM at 45: Enhancements in Accessibility, Functionality, and Speed. J. Phys. Chem. B.

[66] Brooks B.R., Brooks C.L., MacKerell A.D., Nilsson L., Petrella R.J., Roux B., Won Y., Archontis G., Bartels C., Boresch S. (2009). CHARMM: The Biomolecular Simulation Program. J. Comput. Chem..

[67] Jorgensen W.L., Chandrasekhar J., Madura J.D., Impey R.W., Klein M.L. (1983). Comparison of Simple Potential Functions for Simulating Liquid Water. J. Chem. Phys..

[68] Durell S.R., Brooks B.R., Ben-Naim A. (1994). Solvent-Induced Forces between Two Hydrophilic Groups. J. Phys. Chem..

[69] Beglov D., Roux B. (1994). Finite Representation of an Infinite Bulk System: Solvent Boundary Potential for Computer Simulations. J. Chem. Phys..

[70] Venable R.M., Luo Y., Gawrisch K., Roux B., Pastor R.W. (2013). Simulations of Anionic Lipid Membranes: Development of Interaction-Specific Ion Parameters and Validation Using NMR Data. J. Phys. Chem. B.

[71] Phillips J.C., Hardy D.J., Maia J.D.C., Stone J.E., Ribeiro J.V., Bernardi R.C., Buch R., Fiorin G., Hénin J., Jiang W. (2020). Scalable Molecular Dynamics on CPU and GPU Architectures with NAMD. J. Chem. Phys..

[72] Allen M.P., Tildesley D.J. (2017). Computer Simulation of Liquids.

[73] Steinbach P.J., Brooks B.R. (1994). New Spherical-Cutoff Methods for Long-Range Forces in Macromolecular Simulation. J. Comput. Chem..

[74] Shirts M.R., Mobley D.L., Chodera J.D., Pande V.S. (2007). Accurate and Efficient Corrections for Missing Dispersion Interactions in Molecular Simulations. J. Phys. Chem. B.

[75] Darden T., York D., Pedersen L. (1993). Particle Mesh Ewald: An N·log(N) Method for Ewald Sums in Large Systems. J. Chem. Phys..

[76] Ryckaert J.P., Ciccotti G., Berendsen H.J.C. (1977). Numerical Integration of Cartesian Equations of Motion of a System with Constraints: Molecular Dynamics of n-Alkanes. J. Comput. Phys..

[77] Miyamoto S., Kollman P.A. (1992). Settle: An Analytical Version of the SHAKE and RATTLE Algorithm for Rigid Water Models. J. Comput. Chem..

[78] Martyna G.J., Tobias D.J., Klein M.L. (1994). Constant Pressure Molecular Dynamics Algorithms. J. Chem. Phys..

[79] Feller S.E., Zhang Y.H., Pastor R.W., Brooks B.R. (1995). Constant Pressure Molecular Dynamics Simulation: The Langevin Piston Method. J. Chem. Phys..

[80] Allen M.P., Tildesley D.J. (1987). Computer Simulation of Liquids.

[81] Fiorin G., Klein M.L., Hénin J. (2013). Using Collective Variables to Drive Molecular Dynamics Simulations. Mol. Phys..

[82] Hénin J., Fiorin G., Chipot C., Klein M.L. (2011). Exploring Multidimensional Free Energy Landscapes Using Time-Dependent Biases on Collective Variables. J. Chem. Theory Comput..

[83] Hénin J. (2021). Fast and Accurate Multidimensional Free Energy Integration. J. Chem. Theory Comput..

[84] Humphrey W., Dalke A., Schulten K. (1996). VMD: Visual Molecular Dynamics. J. Mol. Graph..

