# Supplementary material for: Iduronate Ring Puckering Effects on Preferred Glycosidic Linkage Conformations in Heparin/Heparan Sulfate and Dermatan Sulfate Disaccharides
Source: Molecules. 2026 Feb 2;31(3):504. doi: 10.3390/molecules31030504 (PMC12899437; doi:10.3390/molecules31030504)

Supporting Information for:

Iduronate ring puckering effects on preferred glycosidic linkage conformations in heparin/heparan sulfate and dermatan sulfate disaccharides

Olgun Guvench [1,*]

[1] Department of Pharmaceutical Sciences and Administration, School of Pharmacy, Westbrook College of Health Professions, University of New England, 716 Stevens Avenue, Portland, ME 04103, USA

* Correspondence: oguvench@une.edu

Table S1. Abbreviations and chemical compositions for heparin/heparan sulfate and for dermatan sulfate disaccharides. Each disaccharide in the "composition" column was simulated with its IdoA[2S] ring restrained to the $^{1}C_{4}$, $^{2}S_{O}$, $B_{3,O}$, or $^{4}C_{1}$ pucker state, and each of these ring-restrained extended-system adaptive biasing force (eABF) molecular dynamics simulations was run in triplicate for a total of 192 (16 compositions x 4 pucker states x triplicate) 200-ns runs.

Disaccharide type	Abbreviation	Composition*
**Heparin/heparan sulfate**	**HS11**	IdoA*α*1–4GlcNS*α*1-*O*-Me
	**HS12**	IdoA2S*α*1–4GlcNS*α*1-*O*-Me
	**HS13**	IdoA2S*α*1–4GlcNS6S*α*1-*O*-Me
	**HS14**	IdoA2S*α*1–4GlcNS3S6S*α*1-*O*-Me
	**HS21**	GlcNS*α*1–4IdoA*α*1-*O*-Me
	**HS22**	GlcNS*α*1–4IdoA2S*α*1-*O*-Me
	**HS23**	GlcNS6S*α*1–4IdoA2S*α*1-*O*-Me
	**HS24**	GlcNS3S6S*α*1–4IdoA2S*α*1-*O*-Me
**Dermatan sulfate**	**DS1a**	IdoA*α*1–3GalNAc4S*β*1-*O*-Me
	**DS1b**	IdoA2S*α*1–3GalNAc4S*β*1-*O*-Me
	**DS1d**	IdoA2S*α*1–3GalNAc6S*β*1-*O*-Me
	**DS1e**	IdoA*α*1–3GalNAc4S6S*β*1-*O*-Me
	**DS2a**	GalNAc4S*β*1–4IdoA*α*1-*O*-Me
	**DS2b**	GalNAc4S*β*1–4IdoA2S*α*1-*O*-Me
	**DS2d**	GalNAc6S*β*1–4IdoA2S*α*1-*O*-Me
	**DS2e**	GalNAc4S6S*β*1–4IdoA*α*1-*O*-Me

* 2S, 3S, and 4S refer to sulfation at the respective carbon atom position as numbered in **Figure 1**. 6S refers to sulfation at carbon atom position 6, which is the exocyclic carbon directly bonded to carbon atom 5.

Table S2. Average (φ, ψ) for $\Delta G(\varphi, \psi)$ = 0 kcal/mol from triplicate eABF simulations as a function of IdoA[2S] pucker state.

Disaccharide	Pucker state*			
	$^{1}C_{4}$	$^{2}S_{O}$	$B_{3,O}$	$^{4}C_{1}$
**HS11**	(-80.0, 129.2) [0.0, 1.4]	(-65.0, 125.8) [0.0, 1.4]	(-85.0, 128.3) [0.0, 1.4]	(-75.0, 117.5) [0.0, 0.0]
**HS12**	(-83.3, 130.0) [1.4, 0.0]	(-62.5, 120.0) [0.0, 0.0]	(-90.8, 80.0) [1.4, 0.0]	(-75.0, 114.2) [0.0, 1.4]
**HS13**	(-85.0, 130.0) [0.0, 0.0]	(-62.5, 121.7) [0.0, 1.4]	(-90.8, 81.7) [1.4, 1.4]	(-73.3, 115.8) [2.9, 1.4]
**HS14**	(-90.0, 142.5) [0.0, 0.0]	(-68.3, 135.0) [1.4, 0.0]	(-121.7, 113.3) [40.5, 27.4]**	(-76.7, 121.7) [1.4, 1.4]
**HS21**	(74.2, 89.2) [1.4, 1.4]	(81.7, 92.5) [1.4, 0.0]	(63.3, 95.8) [1.4, 1.4]	(66.7, 105.0) [1.4, 0.0]
**HS22**	(80.8, 93.3) [1.4, 1.4]	(62.5, 69.2) [0.0, 1.4]	(63.3, 96.7) [1.4, 1.4]	(76.7, 136.7) [1.4, 3.8]
**HS23**	(62.5, 75.8) [17.3, 14.4]**	(62.5, 70.0) [0.0, 0.0]	(63.3, 95.8) [2.9, 1.4]	(74.2, 122.5) [1.4, 10.0]***
**HS24**	(115.8, 109.2) [57.0, 33.9]****	(65.8, 70.0) [1.4, 0.0]	(65.8, 90.8) [1.4, 7.2]	(72.5, 115.0) [0.0, 0.0]
**DS1a**	(-74.2, -105.8) [2.9, 2.9]	(-60.8, -117.5) [1.4, 0.0]	(-71.7, -105.8) [1.4, 1.4]	(-66.7, -119.2) [1.4, 1.4]
**DS1b**	(-74.2, -107.5) [1.4, 0.0]	(-55.0, -120.0) [0.0, 0.0]	(-65.8, -107.5) [1.4, 0.0]	(-70.0, -113.3) [0.0, 1.4]
**DS1d**	(-75.0, -116.7) [0.0, 2.9]	(-54.2, -126.7) [1.4, 2.9]	(-65.0, -110.0) [2.5, 2.5]	(-66.7, -119.2) [1.4, 1.4]
**DS1e**	(-72.5, -107.5) [0.0, 0.0]	(-62.5, -117.5) [0.0, 0.0]	(-72.5, -107.5) [0.0, 0.0]	(-67.5, -117.5) [0.0, 0.0]
**DS2a**	(-60.0, 105.0) [0.0, 0.0]	(-74.2, 100.0) [11.5, 21.7]***	(-77.5, 136.7) [0.0, 1.4]	(-73.3, 154.2) [1.4, 1.4]
**DS2b**	(-60.0, 110.0) [2.5, 4.3]	(-78.3, 111.7) [8.0, 33.9]***	(-76.7, 136.7) [1.4, 1.4]	(-70.8, 156.7) [1.4, 1.4]
**DS2d**	(-69.2, 122.5) [11.3, 15.6]***	(-70.0, 128.3) [2.5, 1.4]	(-75.0, 136.7) [0.0, 1.4]	(-72.5, 157.5) [0.0, 2.5]
**DS2e**	(-65.0, 106.7) [0.0, 2.9]	(-83.3, 79.2) [1.4, 1.4]	(-76.7, 137.5) [1.4, 0.0]	(-72.5, 152.5) [0.0, 0.0]

* "(mean(φ), mean(ψ)) [stdev(φ), stdev(ψ)]" values are in units of degrees. "mean" is the arithmetic mean and "stdev" is the sample standard deviation s from triplicate $\Delta G(\varphi, \psi) = 0$ kcal/mol data with IdoA[2S] restrained to the indicated pucker state. Entries with large s values are in red font.

** Large standard deviation due to two distinct minima with $\Delta G(\varphi, \psi) \approx 0$ kcal/mol in the global-minimum basin.

*** Large standard deviation due to a broad global-minimum basin with a large region of its (φ, ψ)-space having $\Delta G(\varphi, \psi) \approx 0$ kcal/mol.

**** Large standard deviation due to two distinct minima with $\Delta G(\varphi, \psi) \approx 0$ kcal/mol.

Table S3. Differences in (φ, ψ) for $\Delta G(\varphi, \psi) = 0$ kcal/mol as a function of IdoA[2S] pucker state. Difference data, shown in parentheses, are computed from mean values in **Table S2**. Error estimates *SEM*, shown in brackets, are computed from sample standard deviations s in **Table S2** using $SEM = ((s_i^2 + s_j^2) / 3)^{0.5}$, where the indices i and j refer to the pucker states.

hs11	1c4	2so	b3o	4c1
1c4	(0.0, 0.0)	(15.0, -3.3)	(-5.0, -0.8)	(5.0, -11.7)
	[0.0, 1.2]	[0.0, 1.2]	[0.0, 1.2]	[0.0, 0.8]
2so	(-15.0, 3.3)	(0.0, 0.0)	(-20.0, 2.5)	(-10.0, -8.3)
	[0.0, 1.2]	[0.0, 1.2]	[0.0, 1.2]	[0.0, 0.8]
b3o	(5.0, 0.8)	(20.0, -2.5)	(0.0, 0.0)	(10.0, -10.8)
	[0.0, 1.2]	[0.0, 1.2]	[0.0, 1.2]	[0.0, 0.8]
4c1	(-5.0, 11.7)	(10.0, 8.3)	(-10.0, 10.8)	(0.0, 0.0)
	[0.0, 0.8]	[0.0, 0.8]	[0.0, 0.8]	[0.0, 0.0]

hs12	1c4	2so	b3o	4c1
1c4	(0.0, 0.0)	(20.8, -10.0)	(-7.5, -50.0)	(8.3, -15.8)
	[1.2, 0.0]	[0.8, 0.0]	[1.2, 0.0]	[0.8, 0.8]
2so	(-20.8, 10.0)	(0.0, 0.0)	(-28.3, -40.0)	(-12.5, -5.8)
	[0.8, 0.0]	[0.0, 0.0]	[0.8, 0.0]	[0.0, 0.8]
b3o	(7.5, 50.0)	(28.3, 40.0)	(0.0, 0.0)	(15.8, 34.2)
	[1.2, 0.0]	[0.8, 0.0]	[1.2, 0.0]	[0.8, 0.8]
4c1	(-8.3, 15.8)	(12.5, 5.8)	(-15.8, -34.2)	(0.0, 0.0)
	[0.8, 0.8]	[0.0, 0.8]	[0.8, 0.8]	[0.0, 1.2]

hs13	1c4	2so	b3o	4c1
1c4	(0.0, 0.0)	(22.5, -8.3)	(-5.8, -48.3)	(11.7, -14.2)
	[0.0, 0.0]	[0.0, 0.8]	[0.8, 0.8]	[1.7, 0.8]
2so	(-22.5, 8.3)	(0.0, 0.0)	(-28.3, -40.0)	(-10.8, -5.8)
	[0.0, 0.8]	[0.0, 1.2]	[0.8, 1.2]	[1.7, 1.2]
b3o	(5.8, 48.3)	(28.3, 40.0)	(0.0, 0.0)	(17.5, 34.2)
	[0.8, 0.8]	[0.8, 1.2]	[1.2, 1.2]	[1.9, 1.2]
4c1	(-11.7, 14.2)	(10.8, 5.8)	(-17.5, -34.2)	(0.0, 0.0)
	[1.7, 0.8]	[1.7, 1.2]	[1.9, 1.2]	[2.4, 1.2]

hs14	1c4	2so	b3o	4c1
1c4	(0.0, 0.0)	(21.7, -7.5)	(-31.7, -29.2)	(13.3, -20.8)
	[0.0, 0.0]	[0.8, 0.0]	[23.4, 15.8]	[0.8, 0.8]
2so	(-21.7, 7.5)	(0.0, 0.0)	(-53.3, -21.7)	(-8.3, -13.3)
	[0.8, 0.0]	[1.2, 0.0]	[23.4, 15.8]	[1.2, 0.8]
b3o	(31.7, 29.2)	(53.3, 21.7)	(0.0, 0.0)	(45.0, 8.3)
	[23.4, 15.8]	[23.4, 15.8]	[33.1, 22.4]	[23.4, 15.9]
4c1	(-13.3, 20.8)	(8.3, 13.3)	(-45.0, -8.3)	(0.0, 0.0)
	[0.8, 0.8]	[1.2, 0.8]	[23.4, 15.9]	[1.2, 1.2]

hs21	1c4	2so	b3o	4c1
1c4	(0.0, 0.0)	(7.5, 3.3)	(-10.8, 6.7)	(-7.5, 15.8)
	[1.2, 1.2]	[1.2, 0.8]	[1.2, 1.2]	[1.2, 0.8]
2so	(-7.5, -3.3)	(0.0, 0.0)	(-18.3, 3.3)	(-15.0, 12.5)
	[1.2, 0.8]	[1.2, 0.0]	[1.2, 0.8]	[1.2, 0.0]
b3o	(10.8, -6.7)	(18.3, -3.3)	(0.0, 0.0)	(3.3, 9.2)
	[1.2, 1.2]	[1.2, 0.8]	[1.2, 1.2]	[1.2, 0.8]
4c1	(7.5, -15.8)	(15.0, -12.5)	(-3.3, -9.2)	(0.0, 0.0)
	[1.2, 0.8]	[1.2, 0.0]	[1.2, 0.8]	[1.2, 0.0]

hs22	1c4	2so	b3o	4c1
1c4	(0.0, 0.0)	(-18.3, -24.2)	(-17.5, 3.3)	(-4.2, 43.3)
	[1.2, 1.2]	[0.8, 1.2]	[1.2, 1.2]	[1.2, 2.4]
2so	(18.3, 24.2)	(0.0, 0.0)	(0.8, 27.5)	(14.2, 67.5)
	[0.8, 1.2]	[0.0, 1.2]	[0.8, 1.2]	[0.8, 2.4]
b3o	(17.5, -3.3)	(-0.8, -27.5)	(0.0, 0.0)	(13.3, 40.0)
	[1.2, 1.2]	[0.8, 1.2]	[1.2, 1.2]	[1.2, 2.4]
4c1	(4.2, -43.3)	(-14.2, -67.5)	(-13.3, -40.0)	(0.0, 0.0)
	[1.2, 2.4]	[0.8, 2.4]	[1.2, 2.4]	[1.2, 3.1]

hs23	1c4	2so	b3o	4c1
1c4	(0.0, 0.0)	(0.0, -5.8)	(0.8, 20.0)	(11.7, 46.7)
	[14.1, 11.8]	[10.0, 8.3]	[10.1, 8.4]	[10.0, 10.1]
2so	(0.0, 5.8)	(0.0, 0.0)	(0.8, 25.8)	(11.7, 52.5)
	[10.0, 8.3]	[0.0, 0.0]	[1.7, 0.8]	[0.8, 5.8]
b3o	(-0.8, -20.0)	(-0.8, -25.8)	(0.0, 0.0)	(10.8, 26.7)
	[10.1, 8.4]	[1.7, 0.8]	[2.4, 1.2]	[1.9, 5.8]
4c1	(-11.7, -46.7)	(-11.7, -52.5)	(-10.8, -26.7)	(0.0, 0.0)
	[10.0, 10.1]	[0.8, 5.8]	[1.9, 5.8]	[1.2, 8.2]

hs24	1c4	2so	b3o	4c1
1c4	(0.0, 0.0)	(-50.0, -39.2)	(-50.0, -18.3)	(-43.3, 5.8)
	[46.6, 27.7]	[32.9, 19.6]	[32.9, 20.0]	[32.9, 19.6]
2so	(50.0, 39.2)	(0.0, 0.0)	(0.0, 20.8)	(6.7, 45.0)
	[32.9, 19.6]	[1.2, 0.0]	[1.2, 4.2]	[0.8, 0.0]
b3o	(50.0, 18.3)	(0.0, -20.8)	(0.0, 0.0)	(6.7, 24.2)
	[32.9, 20.0]	[1.2, 4.2]	[1.2, 5.9]	[0.8, 4.2]
4c1	(43.3, -5.8)	(-6.7, -45.0)	(-6.7, -24.2)	(0.0, 0.0)
	[32.9, 19.6]	[0.8, 0.0]	[0.8, 4.2]	[0.0, 0.0]

ds1a	1c4	2so	b3o	4c1
1c4	(0.0, 0.0)	(13.3, -11.7)	(2.5, 0.0)	(7.5, -13.3)
	[2.4, 2.4]	[1.9, 1.7]	[1.9, 1.9]	[1.9, 1.9]
2so	(-13.3, 11.7)	(0.0, 0.0)	(-10.8, 11.7)	(-5.8, -1.7)
	[1.9, 1.7]	[1.2, 0.0]	[1.2, 0.8]	[1.2, 0.8]
b3o	(-2.5, 0.0)	(10.8, -11.7)	(0.0, 0.0)	(5.0, -13.3)
	[1.9, 1.9]	[1.2, 0.8]	[1.2, 1.2]	[1.2, 1.2]
4c1	(-7.5, 13.3)	(5.8, 1.7)	(-5.0, 13.3)	(0.0, 0.0)
	[1.9, 1.9]	[1.2, 0.8]	[1.2, 1.2]	[1.2, 1.2]

ds1b	1c4	2so	b3o	4c1
1c4	(0.0, 0.0)	(19.2, -12.5)	(8.3, 0.0)	(4.2, -5.8)
	[1.2, 0.0]	[0.8, 0.0]	[1.2, 0.0]	[0.8, 0.8]
2so	(-19.2, 12.5)	(0.0, 0.0)	(-10.8, 12.5)	(-15.0, 6.7)
	[0.8, 0.0]	[0.0, 0.0]	[0.8, 0.0]	[0.0, 0.8]
b3o	(-8.3, 0.0)	(10.8, -12.5)	(0.0, 0.0)	(-4.2, -5.8)
	[1.2, 0.0]	[0.8, 0.0]	[1.2, 0.0]	[0.8, 0.8]
4c1	(-4.2, 5.8)	(15.0, -6.7)	(4.2, 5.8)	(0.0, 0.0)
	[0.8, 0.8]	[0.0, 0.8]	[0.8, 0.8]	[0.0, 1.2]

ds1d	1c4	2so	b3o	4c1
1c4	(0.0, 0.0)	(20.8, -10.0)	(10.0, 6.7)	(8.3, -2.5)
	[0.0, 2.4]	[0.8, 2.4]	[1.4, 2.2]	[0.8, 1.9]
2so	(-20.8, 10.0)	(0.0, 0.0)	(-10.8, 16.7)	(-12.5, 7.5)
	[0.8, 2.4]	[1.2, 2.4]	[1.7, 2.2]	[1.2, 1.9]
b3o	(-10.0, -6.7)	(10.8, -16.7)	(0.0, 0.0)	(-1.7, -9.2)
	[1.4, 2.2]	[1.7, 2.2]	[2.0, 2.0]	[1.7, 1.7]
4c1	(-8.3, 2.5)	(12.5, -7.5)	(1.7, 9.2)	(0.0, 0.0)
	[0.8, 1.9]	[1.2, 1.9]	[1.7, 1.7]	[1.2, 1.2]

ds1e	1c4	2so	b3o	4c1
1c4	(0.0, 0.0)	(10.0, -10.0)	(0.0, 0.0)	(5.0, -10.0)
	[0.0, 0.0]	[0.0, 0.0]	[0.0, 0.0]	[0.0, 0.0]
2so	(-10.0, 10.0)	(0.0, 0.0)	(-10.0, 10.0)	(-5.0, 0.0)
	[0.0, 0.0]	[0.0, 0.0]	[0.0, 0.0]	[0.0, 0.0]
b3o	(0.0, 0.0)	(10.0, -10.0)	(0.0, 0.0)	(5.0, -10.0)
	[0.0, 0.0]	[0.0, 0.0]	[0.0, 0.0]	[0.0, 0.0]
4c1	(-5.0, 10.0)	(5.0, 0.0)	(-5.0, 10.0)	(0.0, 0.0)

	[0.0, 0.0]	[0.0, 0.0]	[0.0, 0.0]	[0.0, 0.0]

ds2a	1c4	2so	b3o	4c1
1c4	(0.0, 0.0)	(-14.2, -5.0)	(-17.5, 31.7)	(-13.3, 49.2)
	[0.0, 0.0]	[6.7, 12.5]	[0.0, 0.8]	[0.8, 0.8]
2so	(14.2, 5.0)	(0.0, 0.0)	(-3.3, 36.7)	(0.8, 54.2)
	[6.7, 12.5]	[9.4, 17.7]	[6.7, 12.5]	[6.7, 12.5]
b3o	(17.5, -31.7)	(3.3, -36.7)	(0.0, 0.0)	(4.2, 17.5)
	[0.0, 0.8]	[6.7, 12.5]	[0.0, 1.2]	[0.8, 1.2]
4c1	(13.3, -49.2)	(-0.8, -54.2)	(-4.2, -17.5)	(0.0, 0.0)
	[0.8, 0.8]	[6.7, 12.5]	[0.8, 1.2]	[1.2, 1.2]

ds2b	1c4	2so	b3o	4c1
1c4	(0.0, 0.0)	(-18.3, 1.7)	(-16.7, 26.7)	(-10.8, 46.7)
	[2.0, 3.5]	[4.9, 19.8]	[1.7, 2.6]	[1.7, 2.6]
2so	(18.3, -1.7)	(0.0, 0.0)	(1.7, 25.0)	(7.5, 45.0)
	[4.9, 19.8]	[6.6, 27.7]	[4.7, 19.6]	[4.7, 19.6]
b3o	(16.7, -26.7)	(-1.7, -25.0)	(0.0, 0.0)	(5.8, 20.0)
	[1.7, 2.6]	[4.7, 19.6]	[1.2, 1.2]	[1.2, 1.2]
4c1	(10.8, -46.7)	(-7.5, -45.0)	(-5.8, -20.0)	(0.0, 0.0)
	[1.7, 2.6]	[4.7, 19.6]	[1.2, 1.2]	[1.2, 1.2]

ds2d	1c4	2so	b3o	4c1
1c4	(0.0, 0.0)	(-0.8, 5.8)	(-5.8, 14.2)	(-3.3, 35.0)
	[9.2, 12.7]	[6.7, 9.1]	[6.5, 9.1]	[6.5, 9.1]
2so	(0.8, -5.8)	(0.0, 0.0)	(-5.0, 8.3)	(-2.5, 29.2)
	[6.7, 9.1]	[2.0, 1.2]	[1.4, 1.2]	[1.4, 1.7]
b3o	(5.8, -14.2)	(5.0, -8.3)	(0.0, 0.0)	(2.5, 20.8)
	[6.5, 9.1]	[1.4, 1.2]	[0.0, 1.2]	[0.0, 1.7]
4c1	(3.3, -35.0)	(2.5, -29.2)	(-2.5, -20.8)	(0.0, 0.0)
	[6.5, 9.1]	[1.4, 1.7]	[0.0, 1.7]	[0.0, 2.0]

ds2e	1c4	2so	b3o	4c1
1c4	(0.0, 0.0)	(-18.3, -27.5)	(-11.7, 30.8)	(-7.5, 45.8)
	[0.0, 2.4]	[0.8, 1.9]	[0.8, 1.7]	[0.0, 1.7]
2so	(18.3, 27.5)	(0.0, 0.0)	(6.7, 58.3)	(10.8, 73.3)
	[0.8, 1.9]	[1.2, 1.2]	[1.2, 0.8]	[0.8, 0.8]
b3o	(11.7, -30.8)	(-6.7, -58.3)	(0.0, 0.0)	(4.2, 15.0)
	[0.8, 1.7]	[1.2, 0.8]	[1.2, 0.0]	[0.8, 0.0]
4c1	(7.5, -45.8)	(-10.8, -73.3)	(-4.2, -15.0)	(0.0, 0.0)
	[0.0, 1.7]	[0.8, 0.8]	[0.8, 0.0]	[0.0, 0.0]

Figure S1. Triplicate ΔG(φ, ψ) data as a function of IdoA pucker. In order, left-to-right, in a row of ΔG(φ, ψ) plots, the IdoA ring is restrained to the $^{1}C_{4}$, $^{2}S_{O}$, $B_{3,O}$, or $^{4}C_{1}$ pucker state. Each column in a panel contains ΔG(φ, ψ) plots from three independent simulations for that particular pucker state. φ is on the x-axis and ψ is on the y-axis, with their values in degrees. ΔG(φ, ψ) is in kcal/mol with contours every 1 kcal/mol from 0–15 kcal/mol, and is colored from blue to red in the range 0–3 kcal/mol. (**a**) **HS11**; (**b**) **HS12**; (**c**) **HS13**; (**d**) **HS14**; (**e**) **HS21**; (**f**) **HS22**; (**g**) **HS23**; (**h**) **HS24**; (**i**) **DS1a**; (**j**) **DS1b**; (**k**) **DS1d**; (**l**) **DS1e**; (**m**) **DS2a**; (**n**) **DS2b**; (**o**) **DS2d**; and (**p**) **DS2e**.

(**a**)

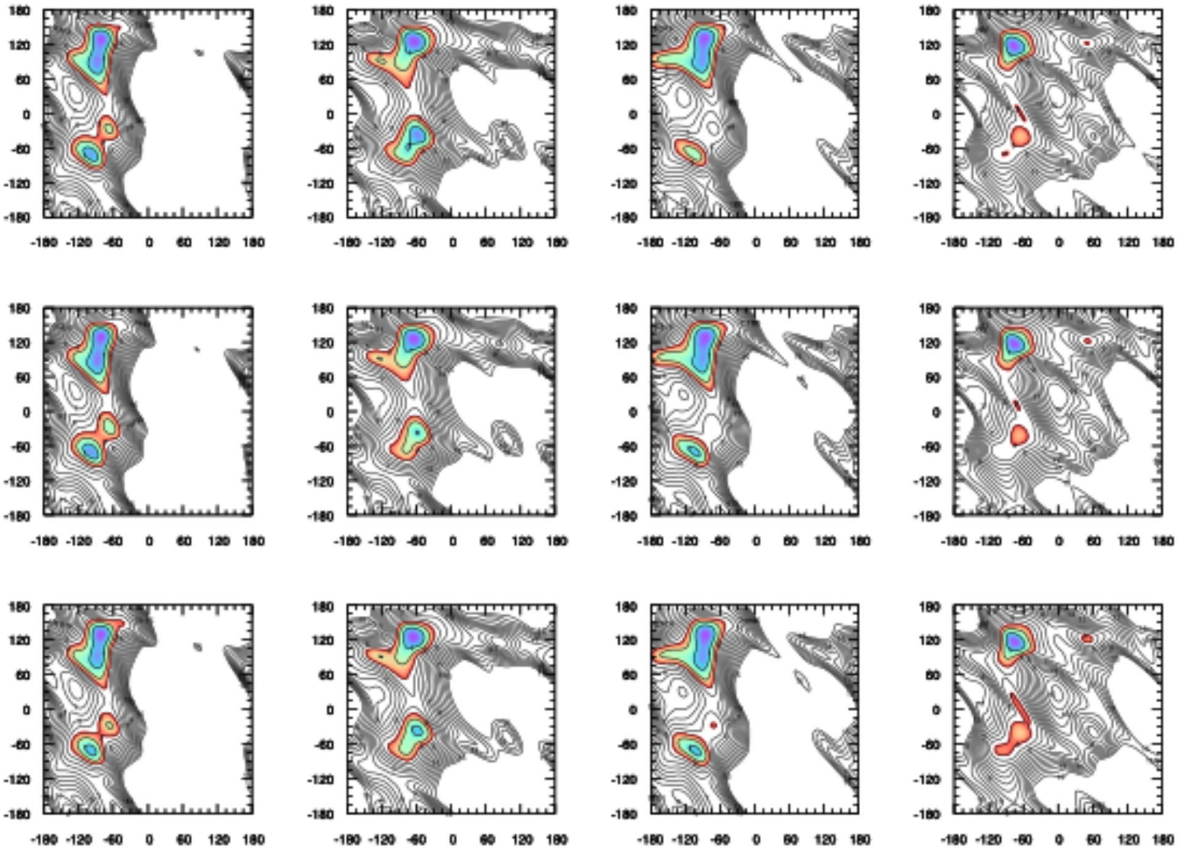

(**b**)

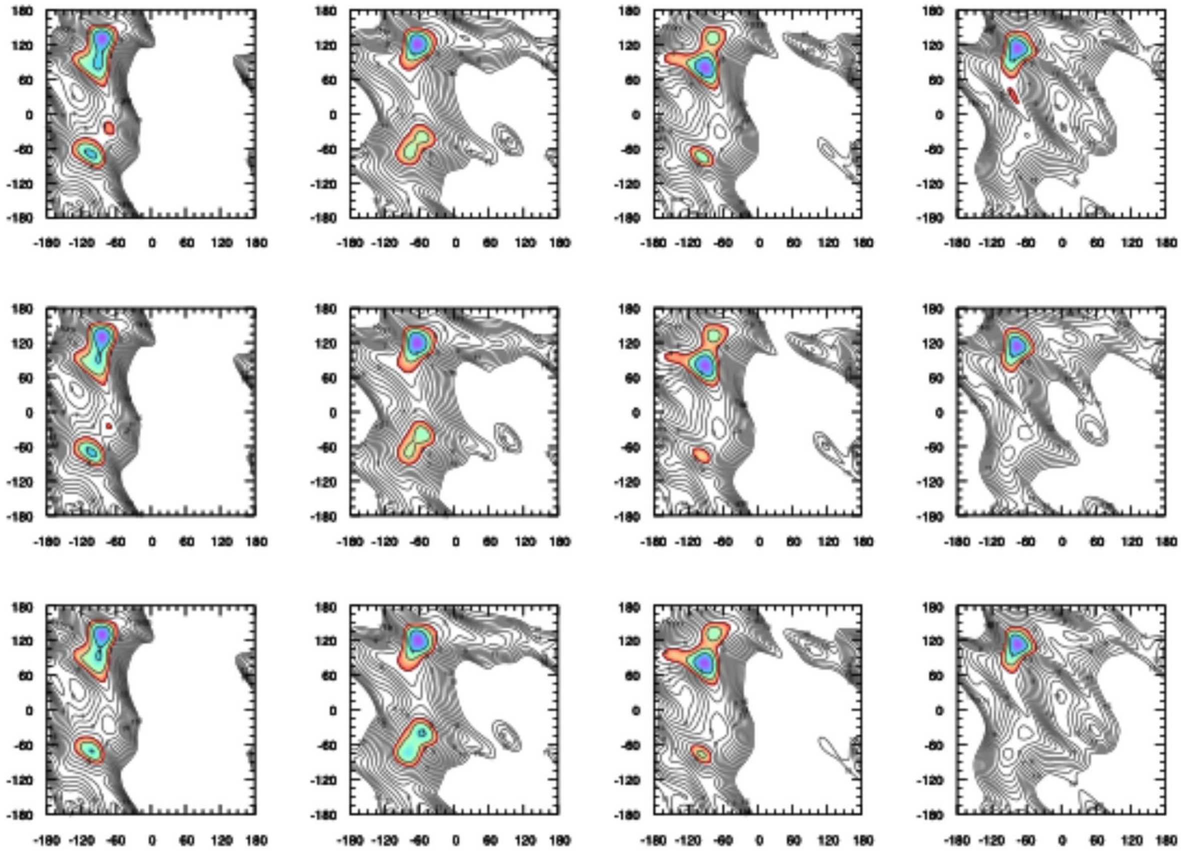

(**c**)

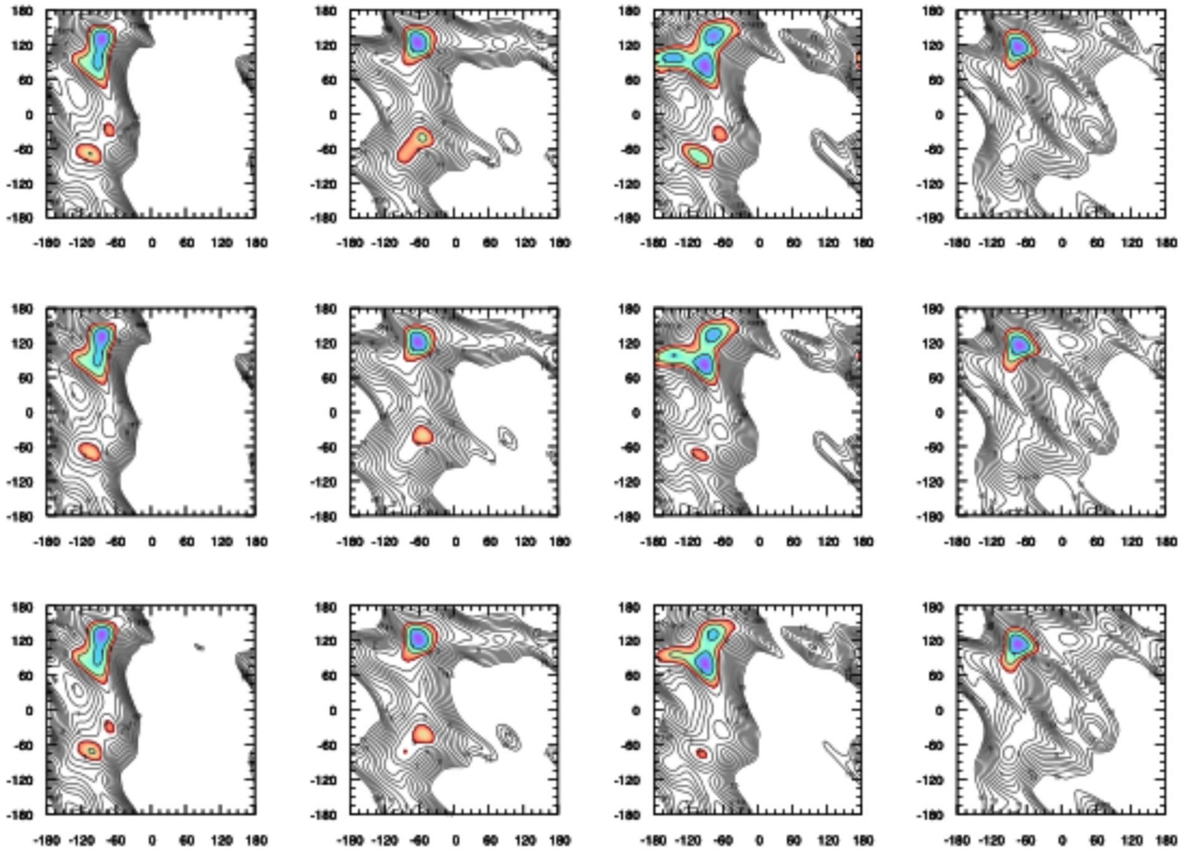

(**d**)

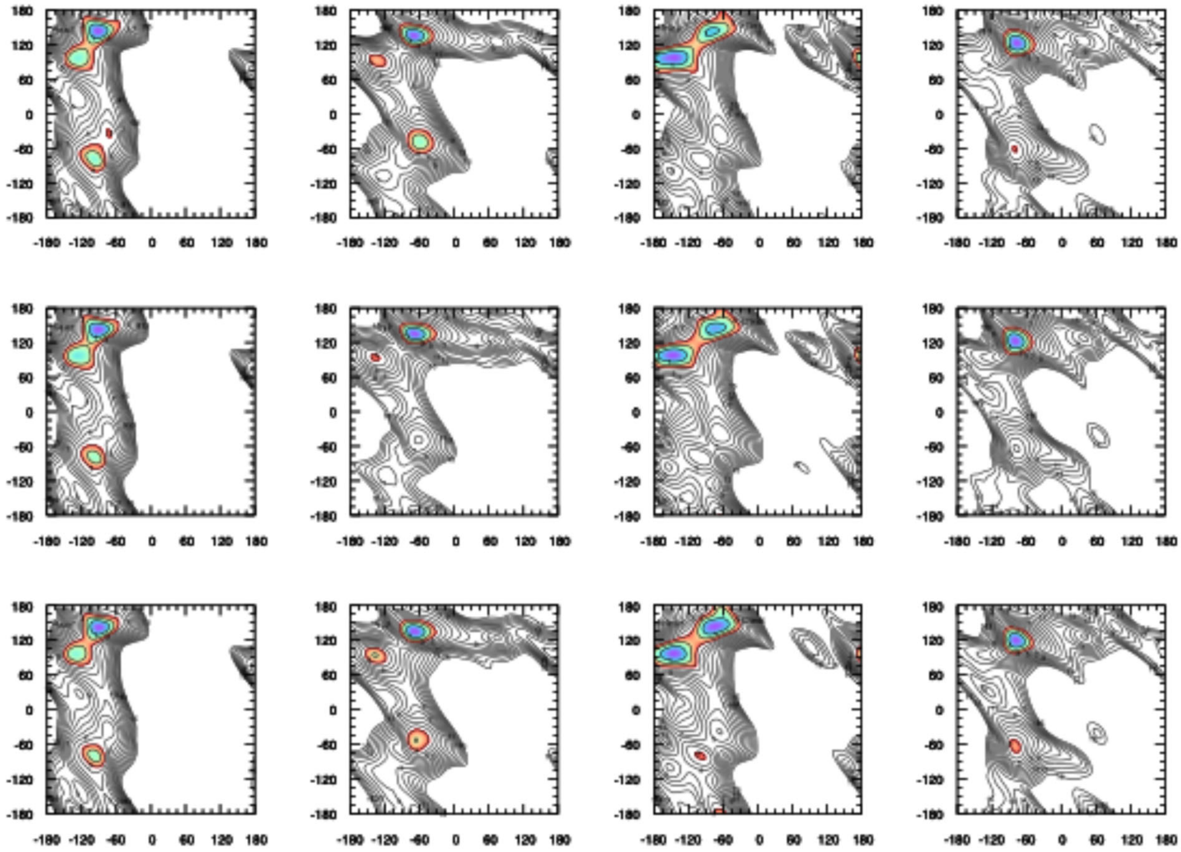

(e)

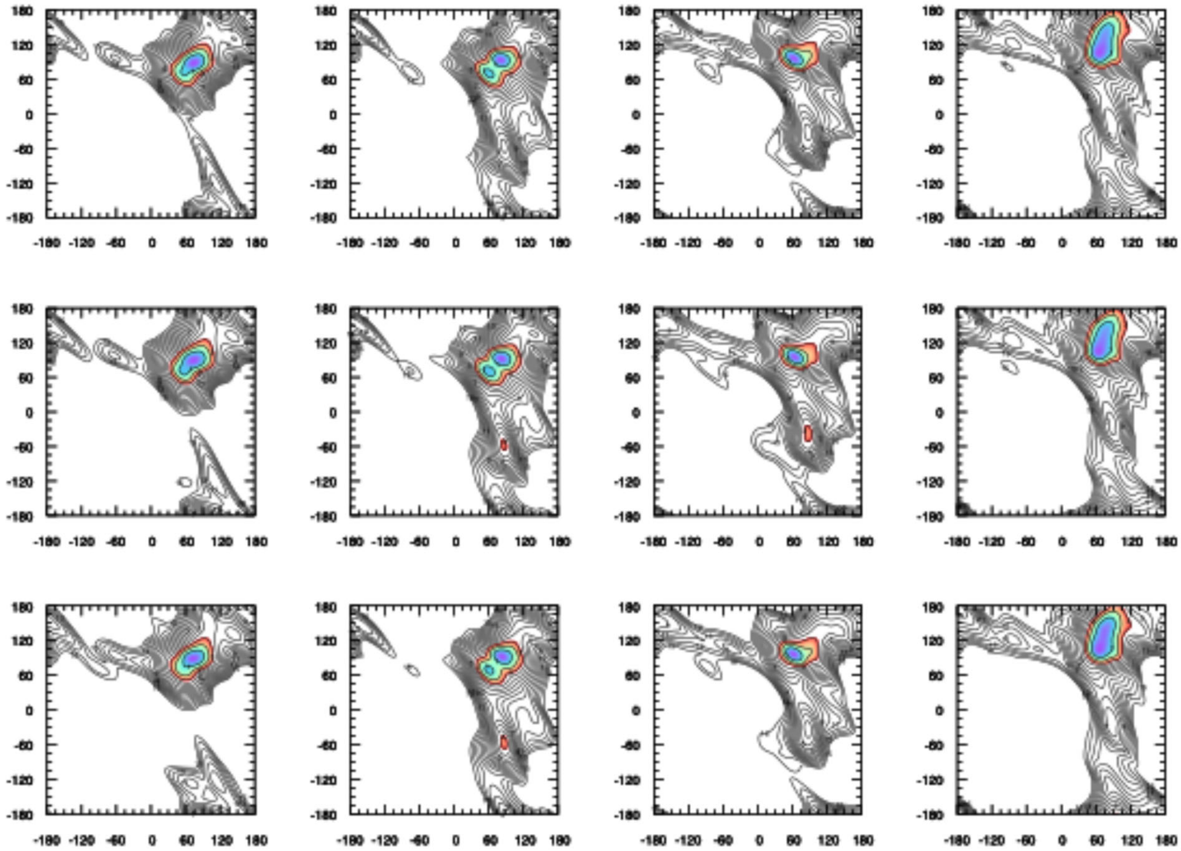

(**f**)

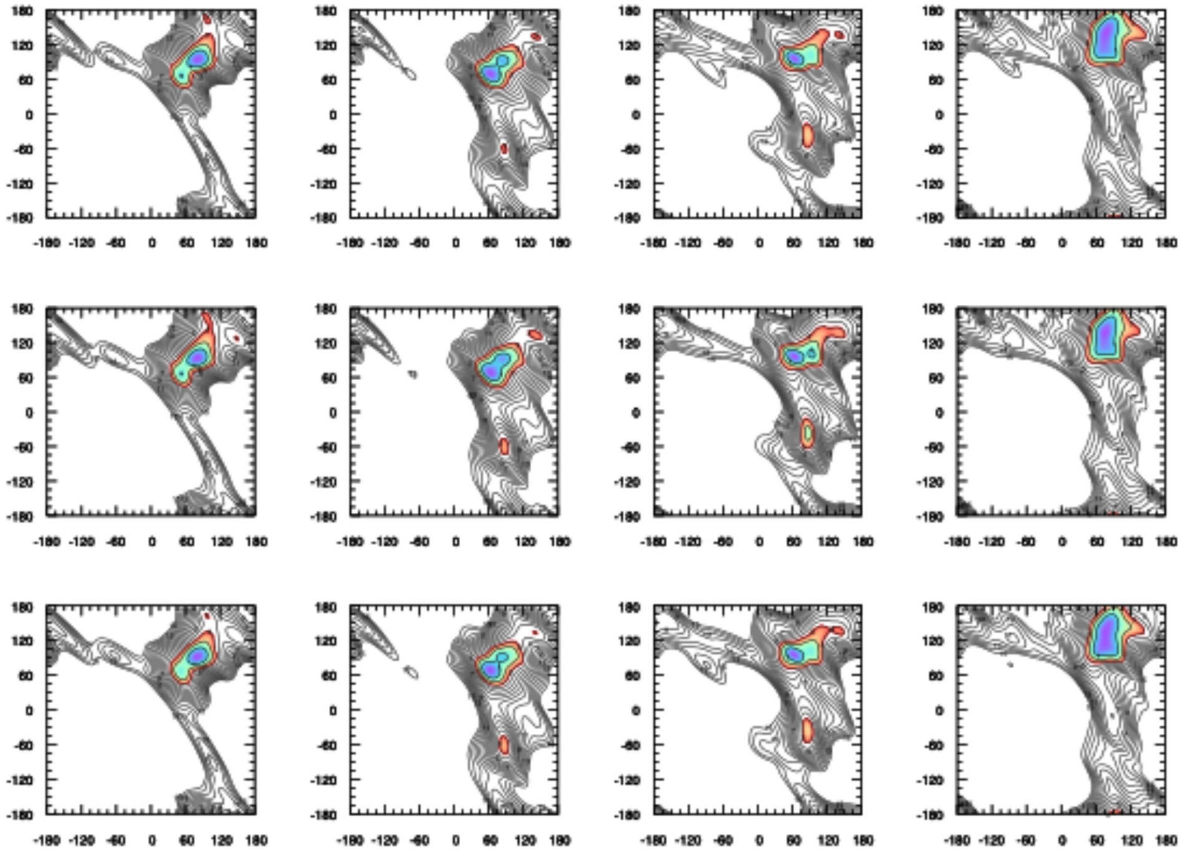

(g)

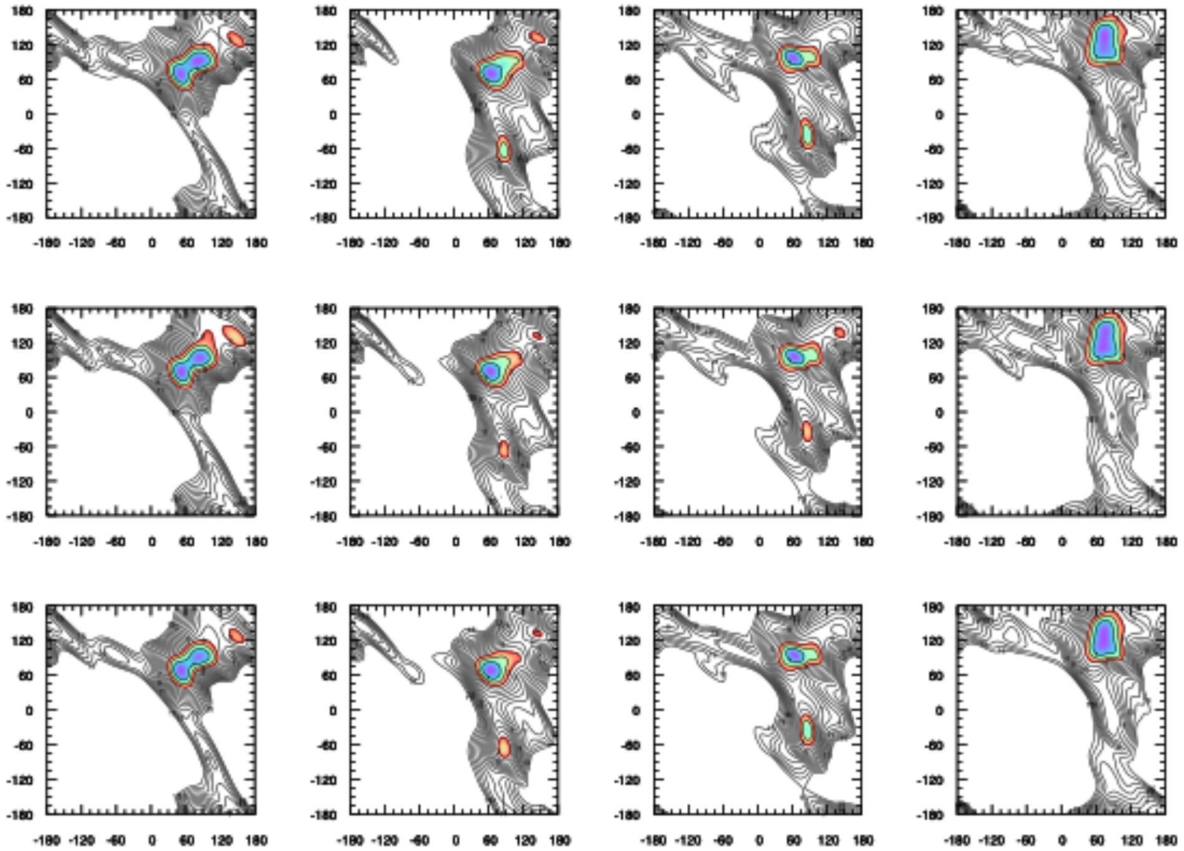

(**h**)

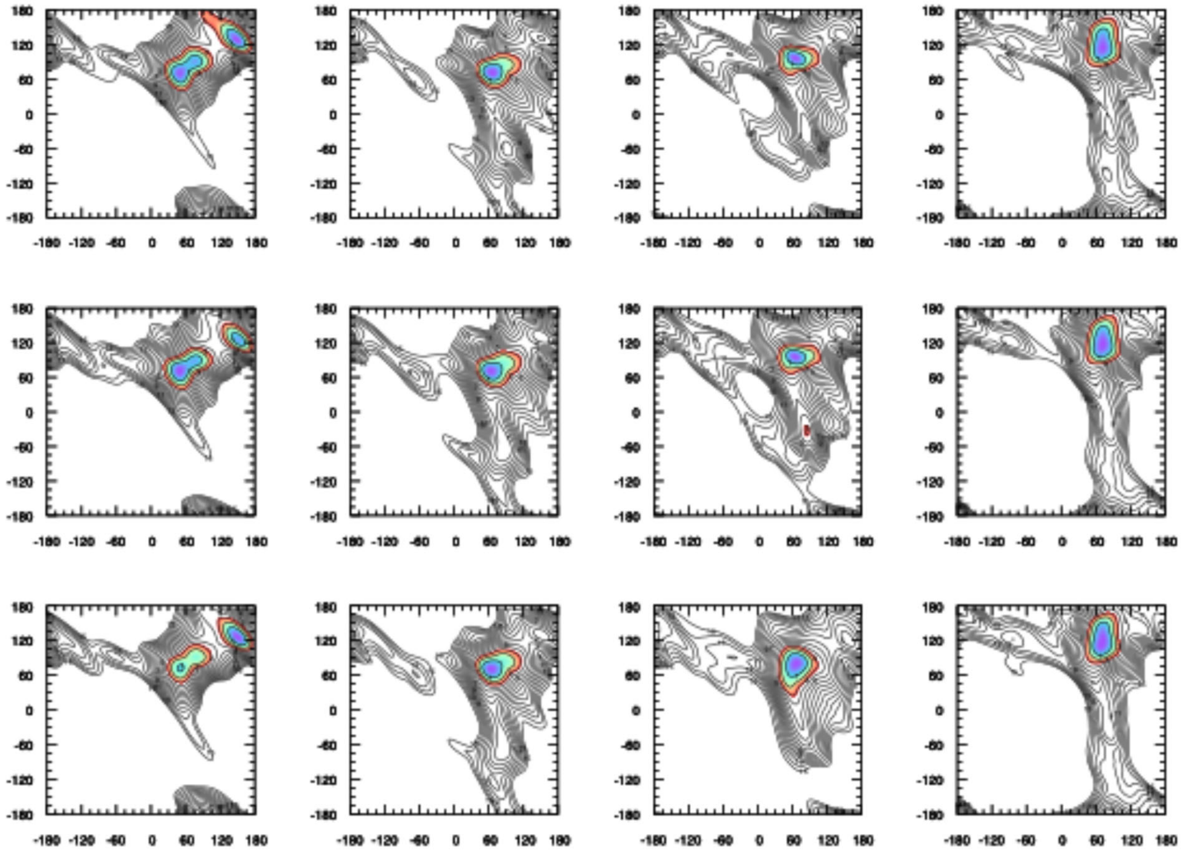

(**i**)

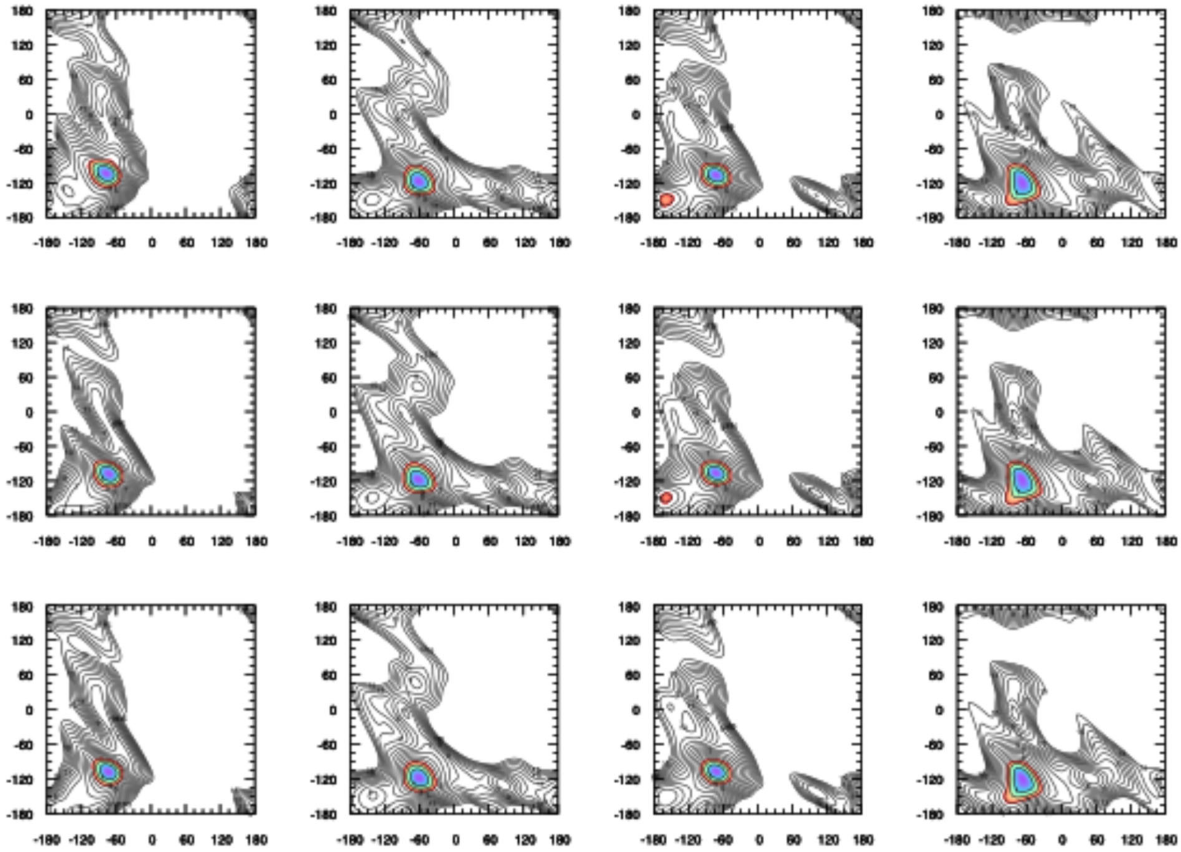

(j)

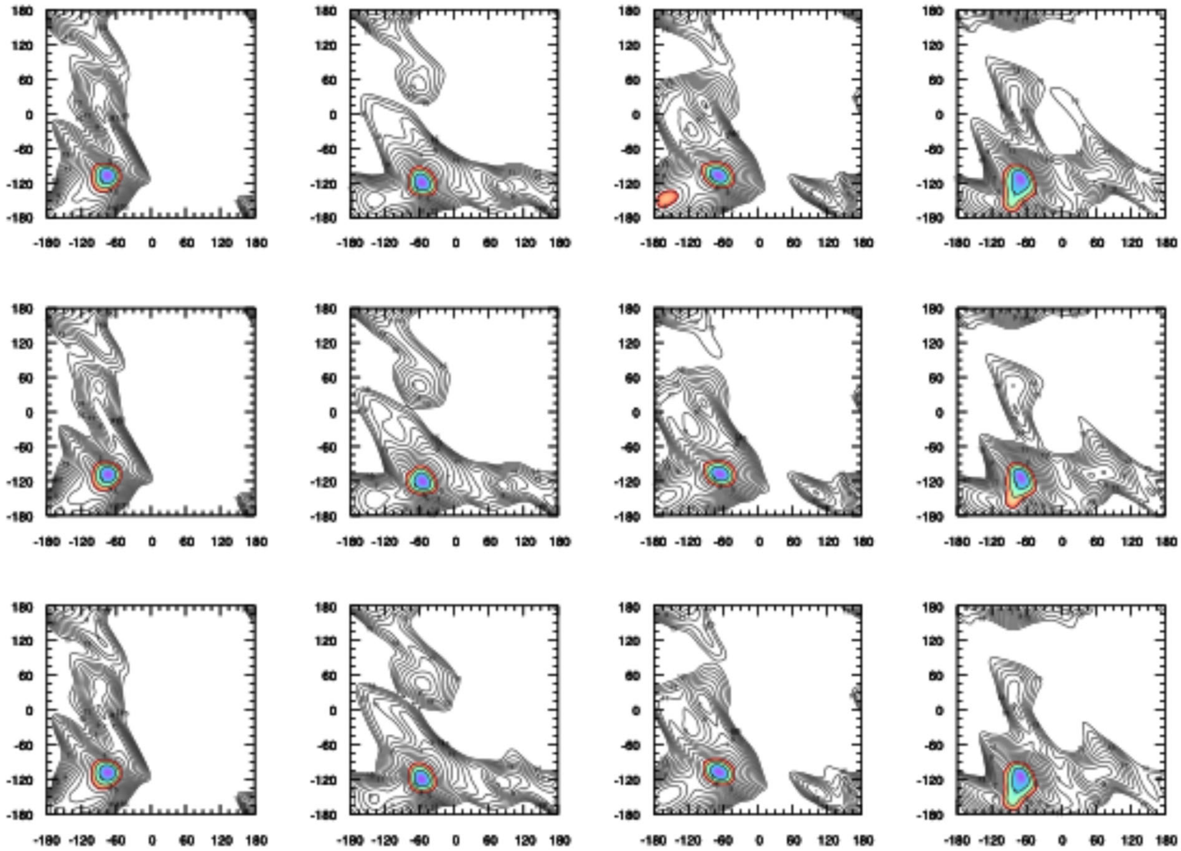

(**k**)

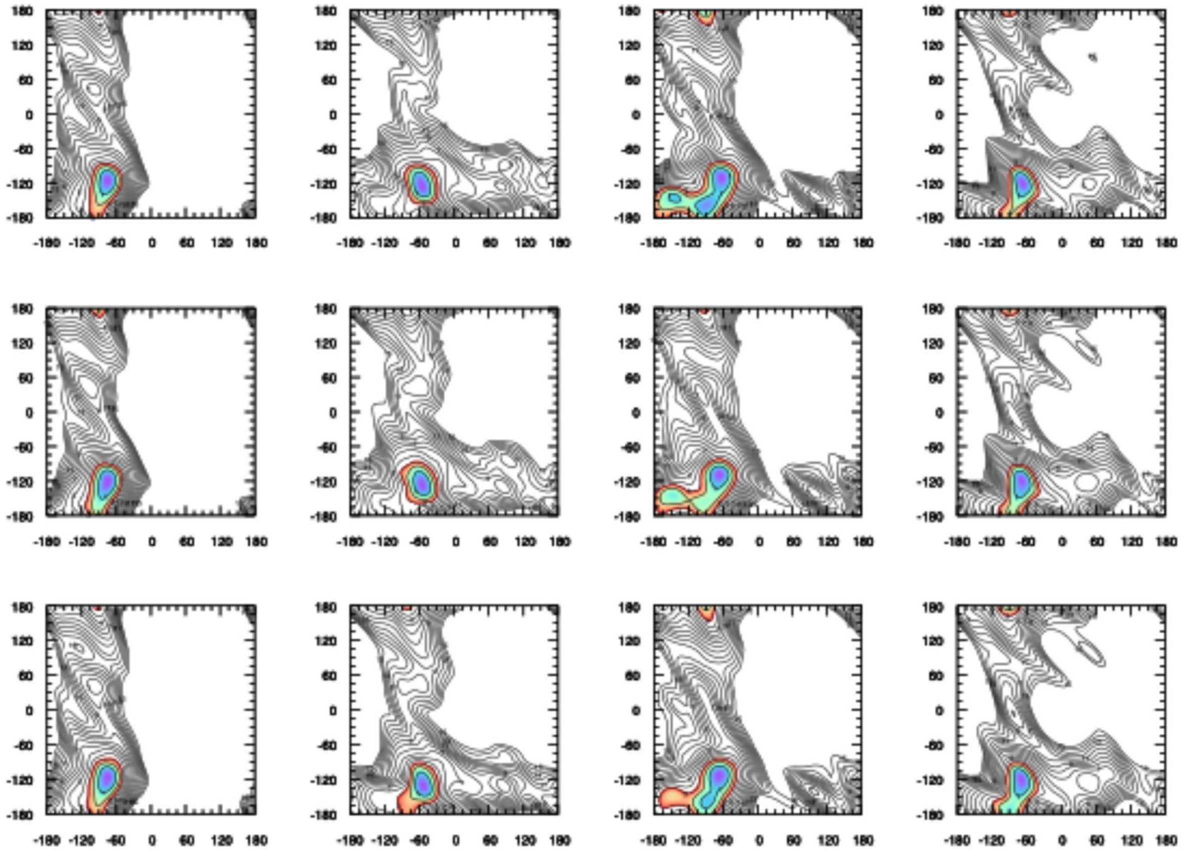

(l)

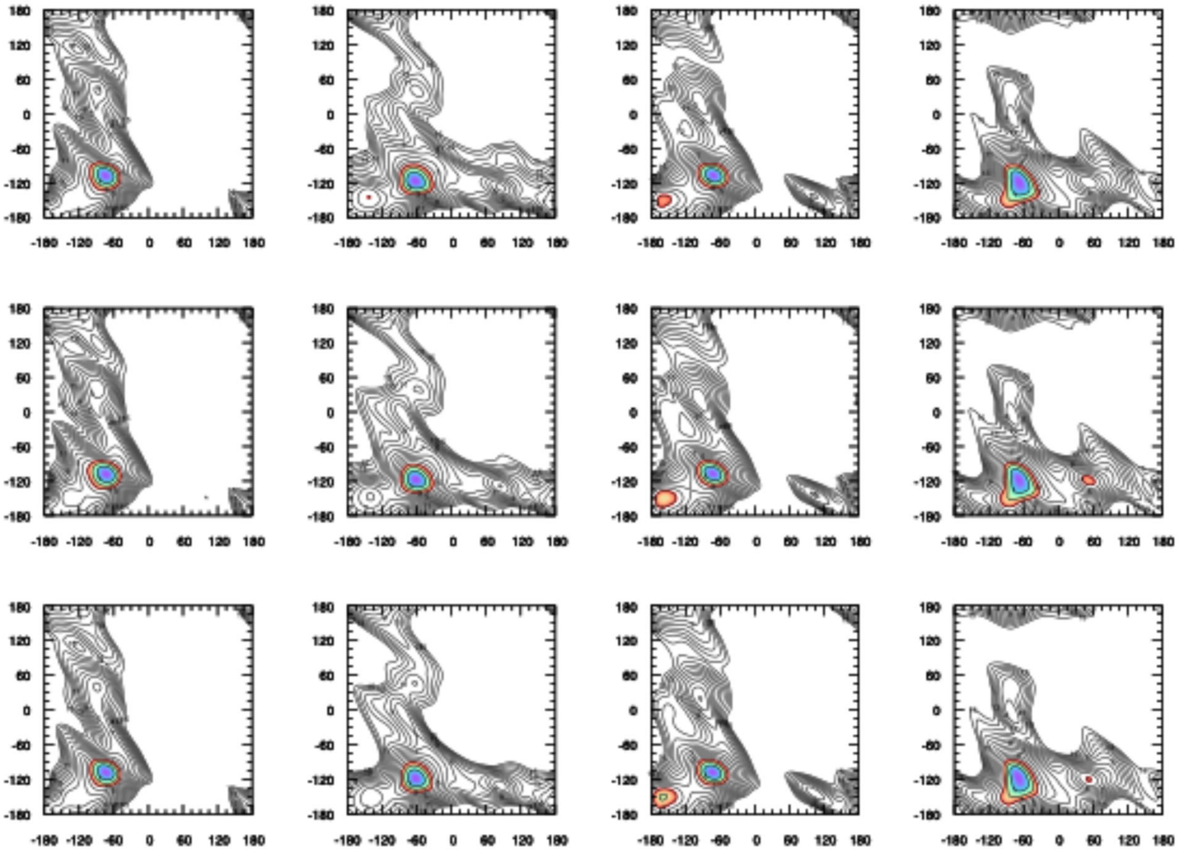

(**m**)

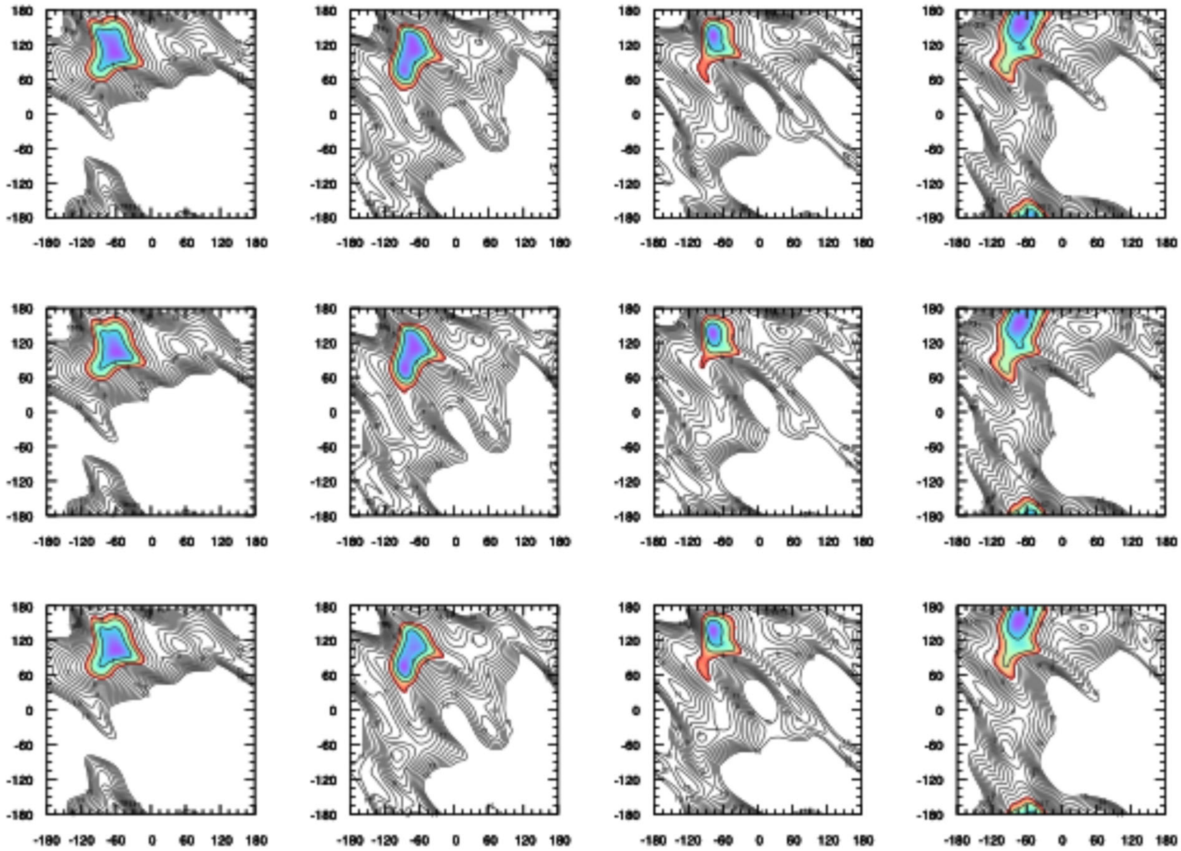

(**n**)

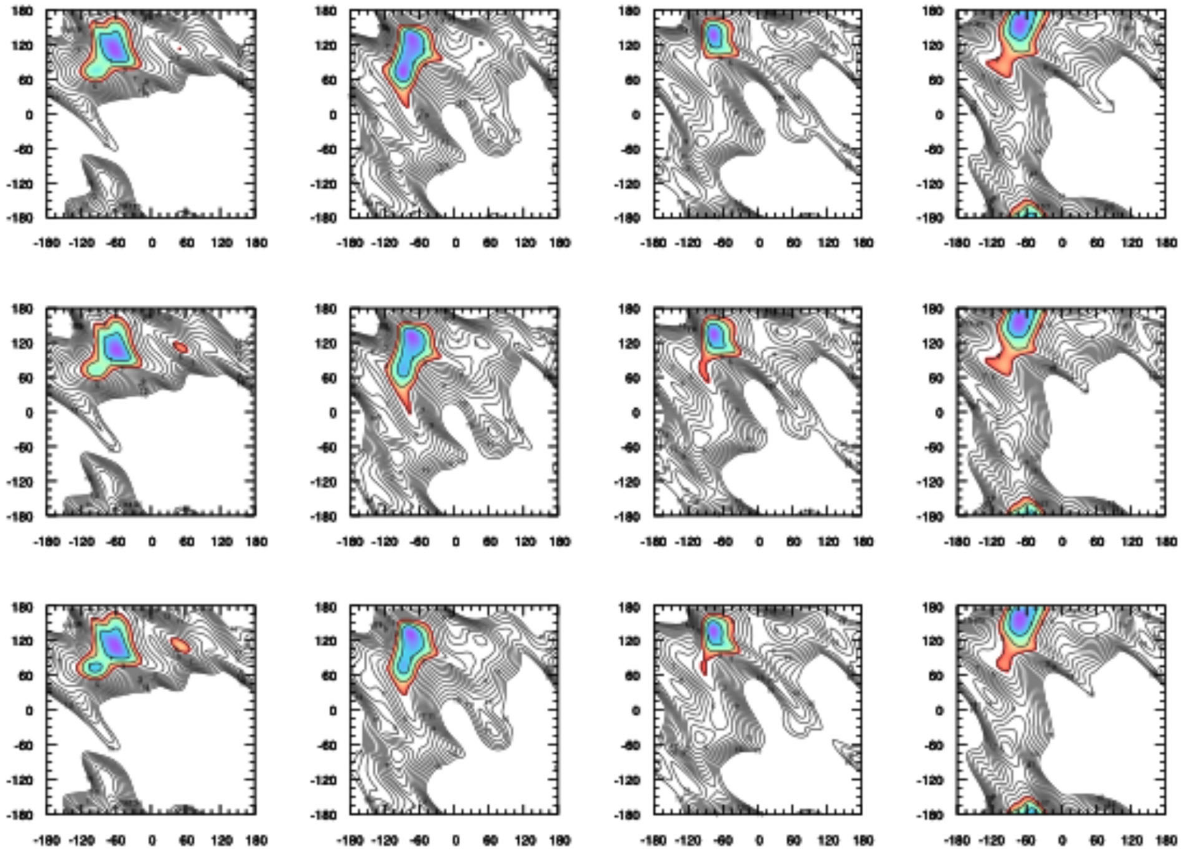

(o)

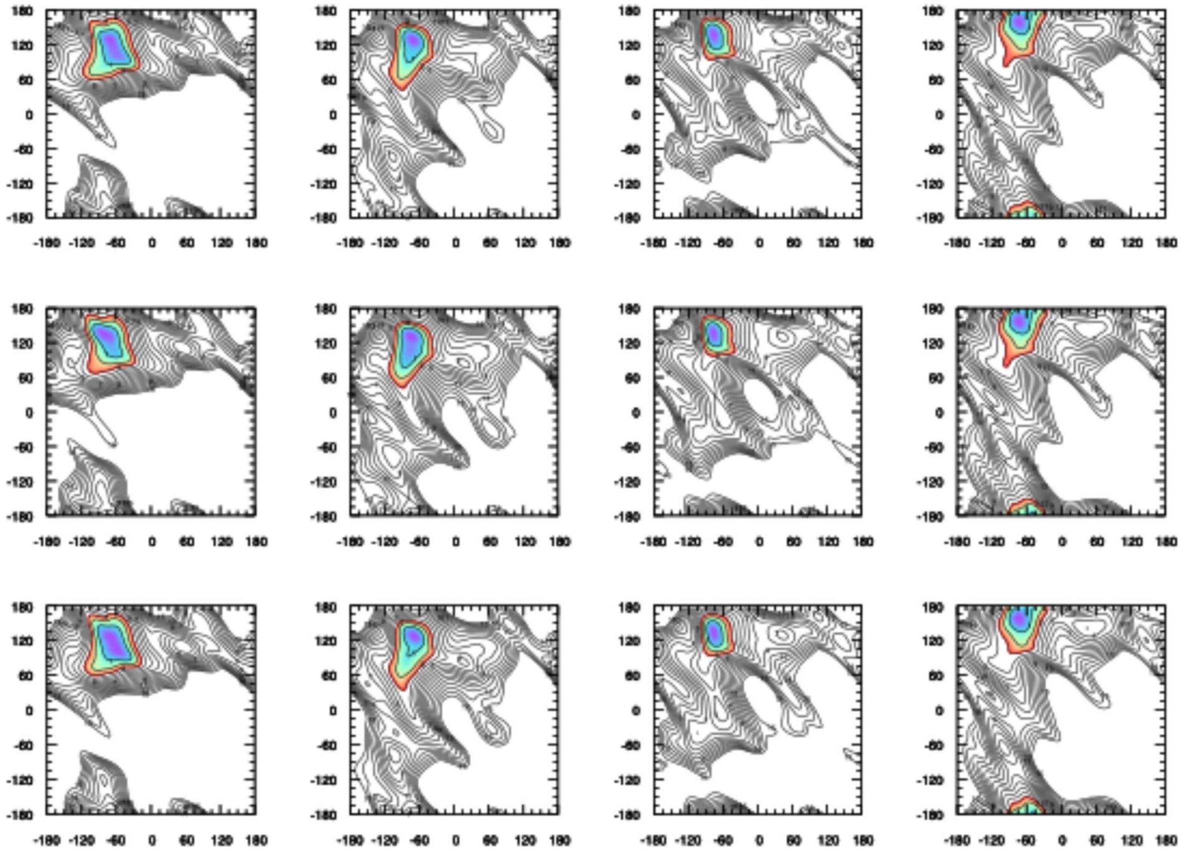

(**p**)

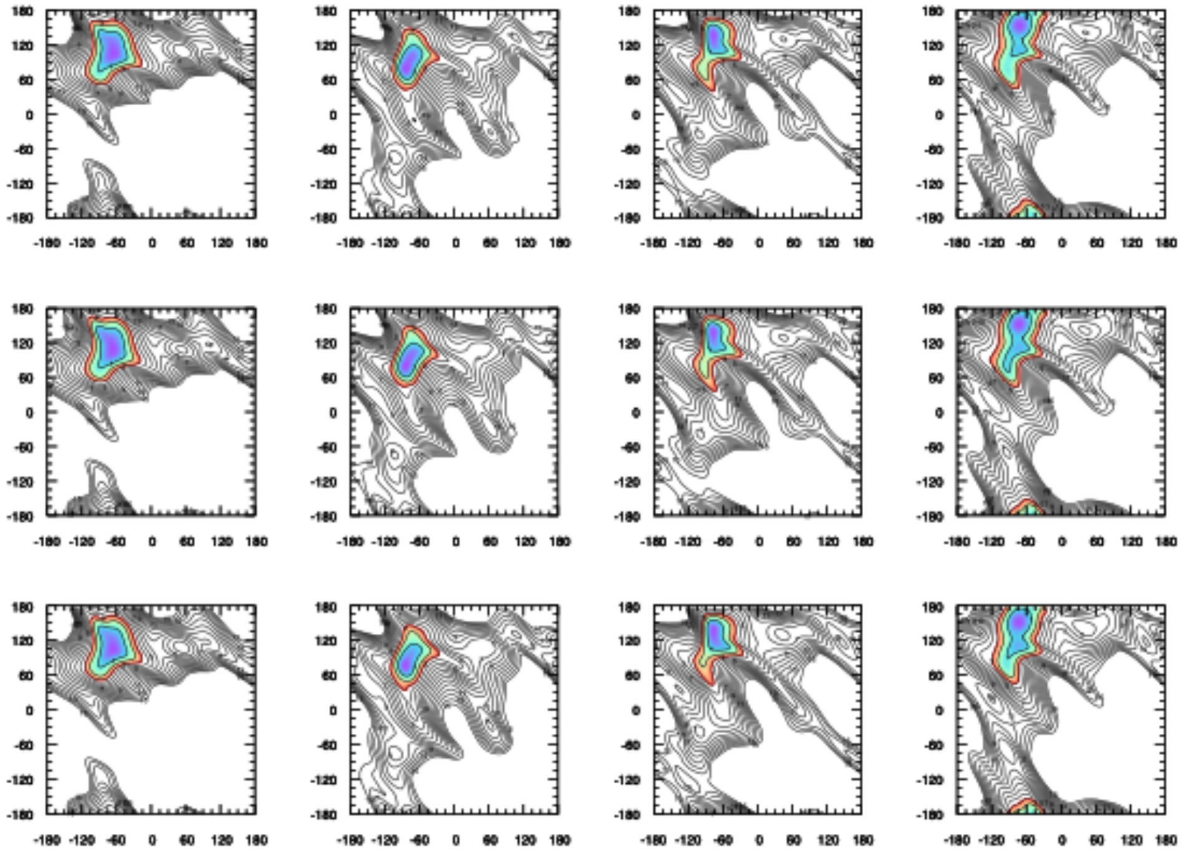

Figure S2. Triplicate Δ*G*(*φ*, *ψ*) data for the IdoA2S*α*1–4GlcNS3S6S glycosidic linkage in GlcNS3S6S*α*1–4IdoA2S*α*1–4GlcNS3S6S*α*1-*O*-Me trisaccharide. eABF simulations were performed as detailed in Methods, with the IdoA2S ring restrained to the $^{2}S_{O}$ pucker state and with the GlcNS3S6S*α*1–4IdoA2S*α*1 (*φ*, *ψ*) set to the global minimum location (65.8°, 70.0°) (**Table S2**: **HS24** $^{2}S_{O}$) for system construction. No biasing or restraints were applied to GlcNS3S6S*α*1–4IdoA2S*α*1 (*φ*, *ψ*) at any time during the eABF simulations.

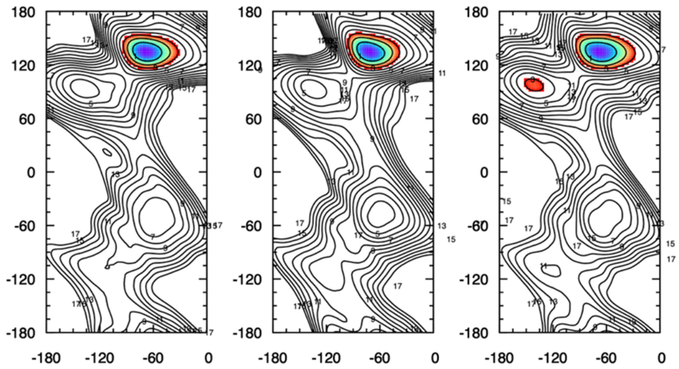

Figure S3. IdoA Cremer–Pople (θ, ϕ) distributions from 200-ns eABF molecular dynamics simulations of **HS11** with the IdoA ring restrained to the $^{1}C_{4}$ (purple), $^{2}S_{O}$ (green), $B_{3,O}$ (blue), or $^{4}C_{1}$ (yellow) pucker state using the ring restraining potential detailed in Methods.

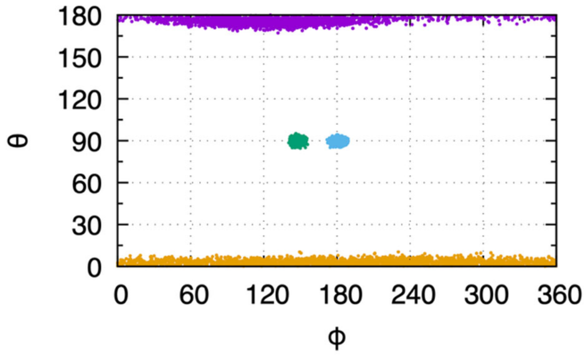

Supplement: Supplementary file 1 [file molecules-31-00504-s001.zip › idoa_disac_phi_psi_v04c_supp.pdf]
